# From Needs Assessment to Instructional Design: Developing the DIF-PBL Model for Inclusive Numeracy Learning

**DOI:** 10.12688/f1000research.187953.1

**Published:** 2026-09-03

**Authors:** Mentari Rama Putri, Arismunandar Arismunandar, Syafruddin Side, Erma Suryani Sahabuddin, Ahmad Swandi

**Affiliations:** 1 Doctoral Program in Educational Sciences, Postgraduate Program,, State University of Makassar, Makassar, South Sulawesi, Indonesia; 2 Educational Administration Study Program, State University of Makassar, Makassar, South Sulawesi, Indonesia; 3 Mathematics Study Program, State University of Makassar, Makassar, South Sulawesi, Indonesia; 4 Population & Environmental Education Study Program, State University of Makassar, Makassar, South Sulawesi, Indonesia; 5 Science Education Study Program, Bosowa University, Makassar, South Sulawesi, Indonesia

**Keywords:** inclusive education; numeracy learning; problem-based learning; differentiated instruction; needs assessment; instructional design.

## Abstract

Inclusive elementary classrooms require instructional approaches that accommodate students with diverse learning characteristics while ensuring equitable opportunities to develop numeracy competencies. Although Problem-Based Learning (PBL) and Differentiated Instruction have individually demonstrated positive educational outcomes, limited studies have translated teachers’ instructional needs into evidence-based design requirements for an integrated instructional model. This study aimed to conduct a comprehensive needs assessment to identify instructional problems, critical root causes, and instructional design requirements that provide the empirical foundation for developing the Differentiation-Based Problem-Based Learning (DIF-PBL) model for inclusive numeracy learning. A mixed-methods convergent parallel design was employed as the initial phase of a Research and Development (R&D) project. Participants included 20 inclusive elementary school teachers who completed a needs assessment questionnaire and three students representing different learner profiles who were involved in initial numeracy observations. Quantitative data were analyzed using descriptive statistics, while qualitative observation data were analyzed descriptively and integrated with quantitative findings through methodological triangulation. The findings revealed four major issues: disparities in students’ numeracy achievement, limitations of conventional PBL in heterogeneous classrooms, practical challenges in implementing differentiated instruction, and a strong consensus regarding the urgency of developing a new instructional model. The analysis identified seven key instructional design requirements: adaptive learning pathways, flexible PBL syntax, Concrete–Pictorial–Abstract (CPA) learning sequences, systematic scaffolding, structured peer tutoring, tiered learning tasks, and adaptive assessment strategies. This study bridges empirical needs assessment and instructional design by translating authentic classroom evidence into evidence-based principles for developing the DIF-PBL model, providing a conceptual and empirical foundation for its subsequent prototype development, validation, and effectiveness evaluation.

## 1. Introduction

Numeracy has become one of the most essential competencies required for individuals to successfully participate in twenty-first-century society ( Rosnelli & Ristiana, 2023). Beyond the ability to perform arithmetic operations, numeracy encompasses the capacity to interpret quantitative information, apply mathematical reasoning, solve contextual problems, and make evidence-based decisions in everyday life. These competencies are increasingly important in an era characterized by rapid technological advancement, data-driven decision-making, and complex societal challenges. Consequently, numeracy is now recognized as a fundamental component of educational quality and a key determinant of students’ future academic achievement, employability, and lifelong learning.

The growing importance of numeracy has been consistently emphasized through international educational assessments. The Programme for International Student Assessment (PISA) defines mathematical literacy as an individual’s ability to formulate, employ, and interpret mathematics in various real-world contexts. Rather than merely measuring procedural calculation, PISA evaluates students’ capacity to reason mathematically, model authentic situations, and solve unfamiliar problems ( OECD, 2022). Despite continuous educational reforms, Indonesian students continue to demonstrate relatively low levels of mathematical literacy ( OECD, 2022). The 2022 PISA results showed that Indonesia’s mathematics performance remained substantially below the OECD average, indicating that many students still struggle to transfer mathematical concepts into meaningful real-life applications. Similar concerns are reflected in the Indonesian Minimum Competency Assessment (Asesmen Kompetensi Minimum/AKM), which identifies numeracy as one of the two core competencies expected of all learners ( Kemendikbudristek, 2021). National education reports consistently indicate that a considerable proportion of elementary school students have not yet achieved the expected level of numeracy proficiency, highlighting the urgent need for instructional innovations capable of improving meaningful mathematical understanding ( Yeh et al., 2019).

These challenges become considerably more complex within inclusive elementary schools. Inclusive education requires students with diverse academic abilities, learning characteristics, and developmental needs to learn together within the same classroom environment ( Moriña, 2017; Shaeffer, 2019). In a typical inclusive classroom, teachers simultaneously accommodate regular students, fast learners, slow learners, and students with special educational needs, each possessing distinct cognitive readiness, learning profiles, communication abilities, and support requirements ( De leon et al., 2024; Jardinez & Natividad, 2024). Such heterogeneity creates substantial instructional challenges because teachers must ensure equitable learning opportunities while maintaining curriculum standards for all students. Consequently, instructional decisions can no longer rely on uniform teaching strategies designed for an “average” learner but must instead accommodate substantial variations in students’ readiness and learning needs.

Numeracy instruction presents particularly significant difficulties in inclusive classrooms because mathematical concepts often progress from concrete experiences toward increasingly abstract representations ( Abdulah et al., 2025; Nair et al., 2024). Students with special educational needs frequently require extended scaffolding, repeated learning experiences, concrete instructional media, and individualized guidance before they are able to understand symbolic mathematical representations ( Evains et al., 2024; Mutodi & Mosimege, 2021; Van Garderen et al., 2018). In contrast, regular students and fast learners generally progress more rapidly through instructional activities. This discrepancy often creates instructional dilemmas in which teachers struggle to divide their attention effectively across students with markedly different learning characteristics ( Goyibova et al., 2025; Smets et al., 2022). As a result, students with special educational needs may experience mathematics anxiety, reduced classroom participation, and persistent learning difficulties, whereas higher-achieving students dominate classroom discussions and collaborative activities. Such conditions frequently widen the achievement gap rather than promoting equitable learning outcomes.

Problem-Based Learning (PBL) has long been recognized as one of the most effective student-centered instructional models for developing higher-order thinking skills, problem-solving ability, collaboration, and conceptual understanding ( Astawan et al., 2025; Suryawati et al., 2020). Through authentic contextual problems, students actively construct knowledge while engaging in inquiry, discussion, and collaborative investigation ( Chin & Chia, 2004; Martawijaya et al., 2023). Numerous studies have demonstrated the positive contribution of PBL to mathematics learning and numeracy development ( Ssali et al., 2025). Consequently, PBL has been widely recommended as an instructional approach capable of fostering meaningful learning experiences that extend beyond procedural knowledge.

However, implementing conventional PBL within inclusive classrooms remains challenging. The characteristics that make PBL effective in general classrooms may also create barriers for students with diverse learning needs. Complex problem scenarios often impose high cognitive demands that exceed the learning readiness of slow learners and students with special educational needs. Open-ended investigations frequently require students to independently regulate learning processes, interpret lengthy contextual information, and participate actively in collaborative discussions ( Emara et al., 2021; Kinnebrew et al., 2017). In heterogeneous groups, fast learners commonly dominate decision-making and task completion, whereas students with special educational needs become passive observers with limited opportunities to contribute meaningfully ( Theobald et al., 2017; Zeng et al., 2024). Furthermore, conventional PBL generally assumes relatively homogeneous student readiness and therefore provides limited instructional flexibility for adapting learning activities, assessment strategies, and teacher support according to individual learning differences. These limitations suggest that although PBL possesses substantial pedagogical potential, its implementation within inclusive classrooms requires systematic instructional adaptation.

Differentiated Instruction has emerged as one of the most promising approaches for addressing learner diversity within inclusive education ( Goyibova et al., 2025; Nketsia et al., 2025). Grounded in the work of Tomlinson, differentiated instruction emphasizes that effective teaching should be responsive to students’ readiness, interests, and learning profiles ( Coubergs et al., 2017; Gheyssens et al., 2022). Rather than providing identical learning experiences for every student, teachers systematically modify instructional content, learning processes, expected products, and classroom environments to ensure that all learners receive appropriate levels of challenge and support. Within numeracy instruction, differentiated teaching enables students to progress according to their individual developmental stages while maintaining common learning objectives.

Despite its considerable potential, differentiated instruction is often difficult to implement consistently in everyday classroom practice. Many teachers experience uncertainty when designing differentiated learning materials, preparing tiered instructional activities, developing adaptive assessments, and managing multiple learning pathways simultaneously. Preparing different lesson plans and instructional resources for diverse student groups substantially increases teachers’ workload and administrative responsibilities. Consequently, differentiation is frequently implemented only partially and remains disconnected from existing instructional models. In particular, previous implementations of differentiated instruction have rarely been systematically integrated with the inquiry-oriented learning processes characteristic of Problem-Based Learning, leaving teachers without a coherent instructional framework specifically designed for inclusive numeracy education.

Previous research has extensively investigated the effectiveness of Problem-Based Learning in improving mathematical achievement and has separately examined the benefits of differentiated instruction for accommodating learner diversity. However, limited studies have systematically explored teachers’ instructional needs as the empirical foundation for developing an integrated Differentiation-Based Problem-Based Learning (DIF-PBL) model specifically designed for inclusive numeracy learning. Existing studies predominantly focus on evaluating instructional effectiveness after model implementation rather than identifying the instructional problems, root causes, and design requirements that should inform the development of an adaptive learning model. As a result, there remains insufficient empirical evidence explaining how teachers’ classroom experiences and instructional challenges can be translated into concrete design principles for inclusive numeracy instruction.

Addressing this gap requires a comprehensive needs assessment that extends beyond identifying teachers’ perceptions of classroom challenges. Such an assessment should examine the factual conditions of numeracy learning, limitations of current Problem-Based Learning implementation, challenges associated with differentiated instruction, students’ initial numeracy characteristics, and the underlying causes of these instructional problems. Understanding these interconnected factors is essential for ensuring that instructional innovations are grounded in authentic classroom realities rather than theoretical assumptions alone.

Based on the literature and the identified research gap, this study proposes a conceptual pathway in which empirical needs assessment serves as the foundation for instructional design. Rather than directly developing a new instructional model from theoretical assumptions, the present study positions teachers’ classroom experiences, students’ learning characteristics, and instructional challenges as the primary sources for deriving design requirements. As illustrated in Figure 1, the needs assessment bridges authentic classroom problems and the conceptual development of the Differentiation-Based Problem-Based Learning (DIF-PBL) model.

**Figure 1.**
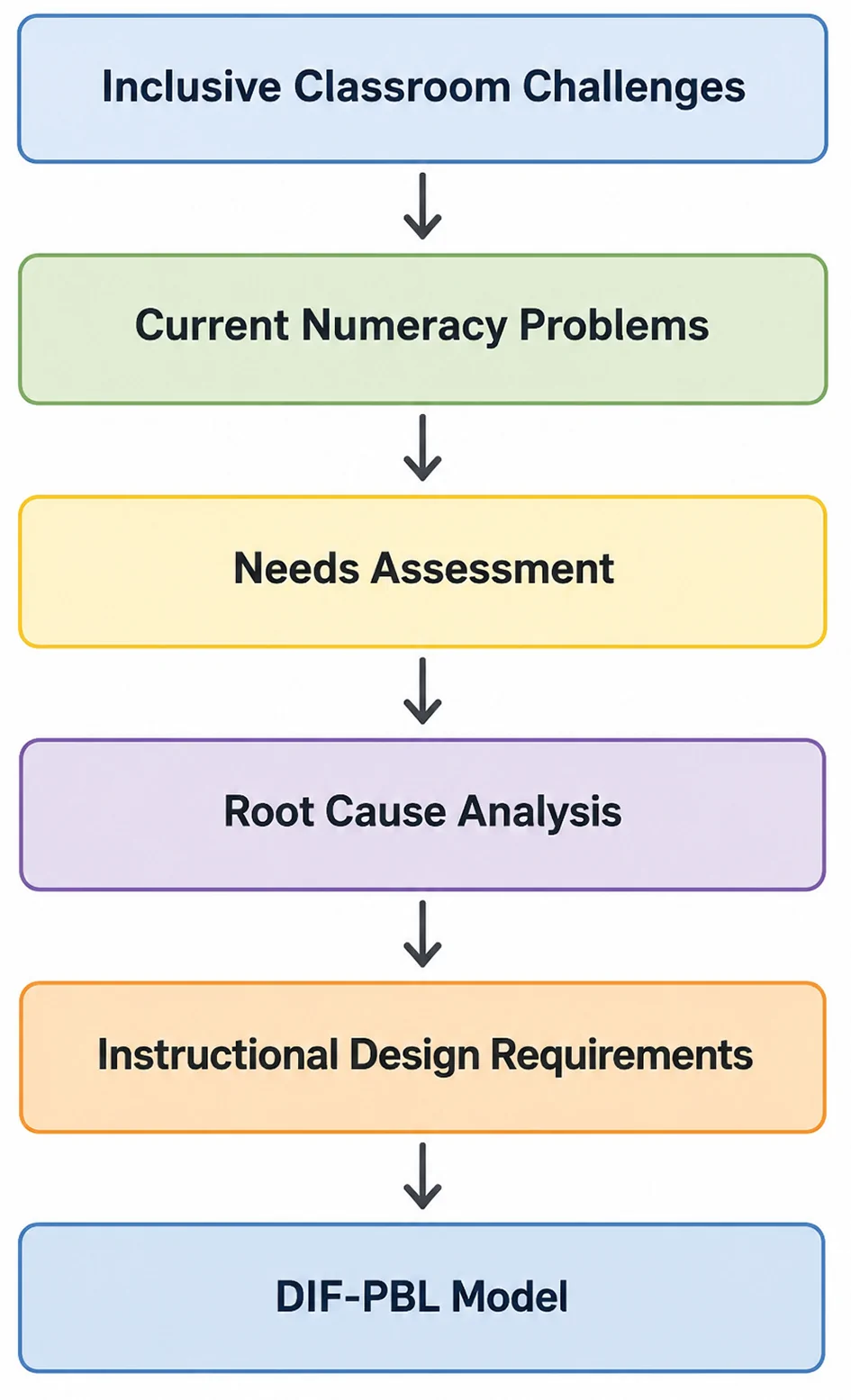
Conceptual framework.

Therefore, this study aimed to conduct a comprehensive needs assessment to identify instructional problems, critical root causes, and instructional design requirements that serve as the empirical foundation for developing the Differentiation-Based Problem-Based Learning (DIF-PBL) model for inclusive elementary schools. The findings are expected to provide evidence-based design principles that guide the subsequent development of an adaptive instructional model capable of supporting equitable, meaningful, and differentiated numeracy learning for students with diverse educational needs. This study addressed the following research questions:

RQ1. What are the current conditions of numeracy learning in inclusive elementary classrooms?

RQ2. What limitations do teachers experience in implementing conventional Problem-Based Learning?

RQ3. What challenges do teachers encounter in implementing differentiated instruction?

RQ4. What instructional design requirements emerge from the comprehensive needs assessment for developing the DIF-PBL model?

## 2. Research methods

### 2.1 Research design

This study represents the initial phase of a Research and Development (R&D) project aimed at developing a Differentiation-Based Problem-Based Learning (DIF-PBL) model for improving numeracy learning in inclusive elementary schools. Specifically, the present study focused on conducting a comprehensive needs assessment to identify instructional problems, teachers’ needs, and design requirements that would serve as the empirical foundation for the subsequent development of the instructional model.

A mixed-methods approach employing a convergent parallel design was adopted to obtain both quantitative and qualitative evidence regarding the current implementation of numeracy instruction in inclusive classrooms. Quantitative data were collected to determine the prevalence of instructional challenges and teachers’ perceptions of their instructional needs, whereas qualitative data were gathered to obtain a deeper understanding of teachers’ classroom experiences and the underlying causes of instructional difficulties. Both types of data were analyzed independently and subsequently integrated during interpretation to provide a comprehensive understanding of the instructional context.

The needs assessment examined four major aspects: (1) the factual conditions of numeracy learning in inclusive classrooms, (2) the implementation of the current Problem-Based Learning (PBL) model, (3) challenges in implementing differentiated instruction, and (4) teachers’ perceptions regarding the urgency of developing a new instructional model. These findings were subsequently synthesized into instructional design requirements that guided the conceptual development of the DIF-PBL model.

### 2.2 Participants

This research was conducted from August 2025 to November 2025. The study was conducted in inclusive elementary schools located in Luwu Regency, South Sulawesi, Indonesia. The research sites were purposively selected because they had implemented inclusive education in which regular students and students with special educational needs learned together in the same classrooms.

Participants consisted of two groups. First, twenty elementary school teachers participated in the questionnaire survey. All teachers were directly involved in teaching mathematics or numeracy in inclusive classrooms and had experience implementing student-centered learning approaches under the Independent Curriculum. Second, three students representing different learner profiles participated in an initial numeracy assessment. These included one regular student, one slow learner, and one student with special educational needs. The inclusion of students with diverse learning characteristics enabled the researchers to obtain an initial description of variations in numeracy performance that informed the instructional design requirements of the proposed model.

### 2.3 Instruments and data collection

Three complementary data collection techniques were employed: questionnaires and classroom-based student observations.

The needs assessment questionnaire was developed to investigate teachers’ perceptions of current numeracy instruction and the necessity of developing a new instructional model. The instrument consisted of thirteen indicators organized into four dimensions: (1) factual conditions of numeracy learning, (2) implementation of Problem-Based Learning, (3) implementation of differentiated instruction, and (4) urgency for developing the DIF-PBL model. Responses were measured using a four-point Likert scale ranging from *strongly disagree* to *strongly agree*, thereby avoiding neutral responses and encouraging participants to express clear judgments regarding each statement.

To complement teachers’ perspectives, an initial observation of students’ numeracy abilities was conducted involving three students with different learning characteristics. Students completed contextual numeracy tasks while researchers observed their approaches to problem solving, use of mathematical representations, need for instructional support, and responses to learning activities. These observations provided preliminary evidence regarding differences in learning characteristics among regular students, slow learners, and students with special educational needs.

The questionnaire was developed based on the theoretical constructs of inclusive numeracy instruction, Problem-Based Learning, and Differentiated Instruction. Prior to data collection, the instrument was reviewed by three experts in mathematics education and inclusive education to evaluate content relevance, clarity, and representativeness. Minor revisions were made based on their recommendations. Internal consistency of the questionnaire was examined using Cronbach’s Alpha, indicating acceptable reliability. Trustworthiness of qualitative findings was strengthened through methodological triangulation involving questionnaires and classroom observations.

### 2.4 Data analysis

Quantitative data obtained from the questionnaires were analyzed using descriptive statistics, including frequencies, percentages, and mean scores. The percentage of responses for each indicator was calculated to identify the relative level of instructional need perceived by teachers. Higher percentages indicated stronger agreement regarding the necessity of instructional improvements and were used to determine priority areas for model development.

Student observation data were analyzed descriptively by comparing patterns of numeracy performance across the three learner profiles. Particular attention was given to differences in conceptual understanding, problem-solving strategies, use of concrete representations, and the degree of instructional assistance required during task completion.

Finally, findings from the questionnaires and student observations were integrated through methodological triangulation to enhance the credibility and comprehensiveness of the needs assessment. Rather than considering each data source independently, the triangulation process enabled the researchers to confirm recurring instructional issues across multiple sources of evidence and to formulate instructional design requirements that informed the conceptual development of the DIF-PBL model.

## 3. Results and discussion

### 3.1 Current conditions of numeracy learning in inclusive classrooms

The first phase of the needs assessment examined teachers’ perceptions of the factual conditions of numeracy learning in inclusive elementary classrooms. Four indicators were evaluated, covering disparities in numeracy achievement, teachers’ instructional challenges, students’ responses to abstract mathematical concepts, and assessment practices. The results are presented in Figure 2.

**Figure 2.**
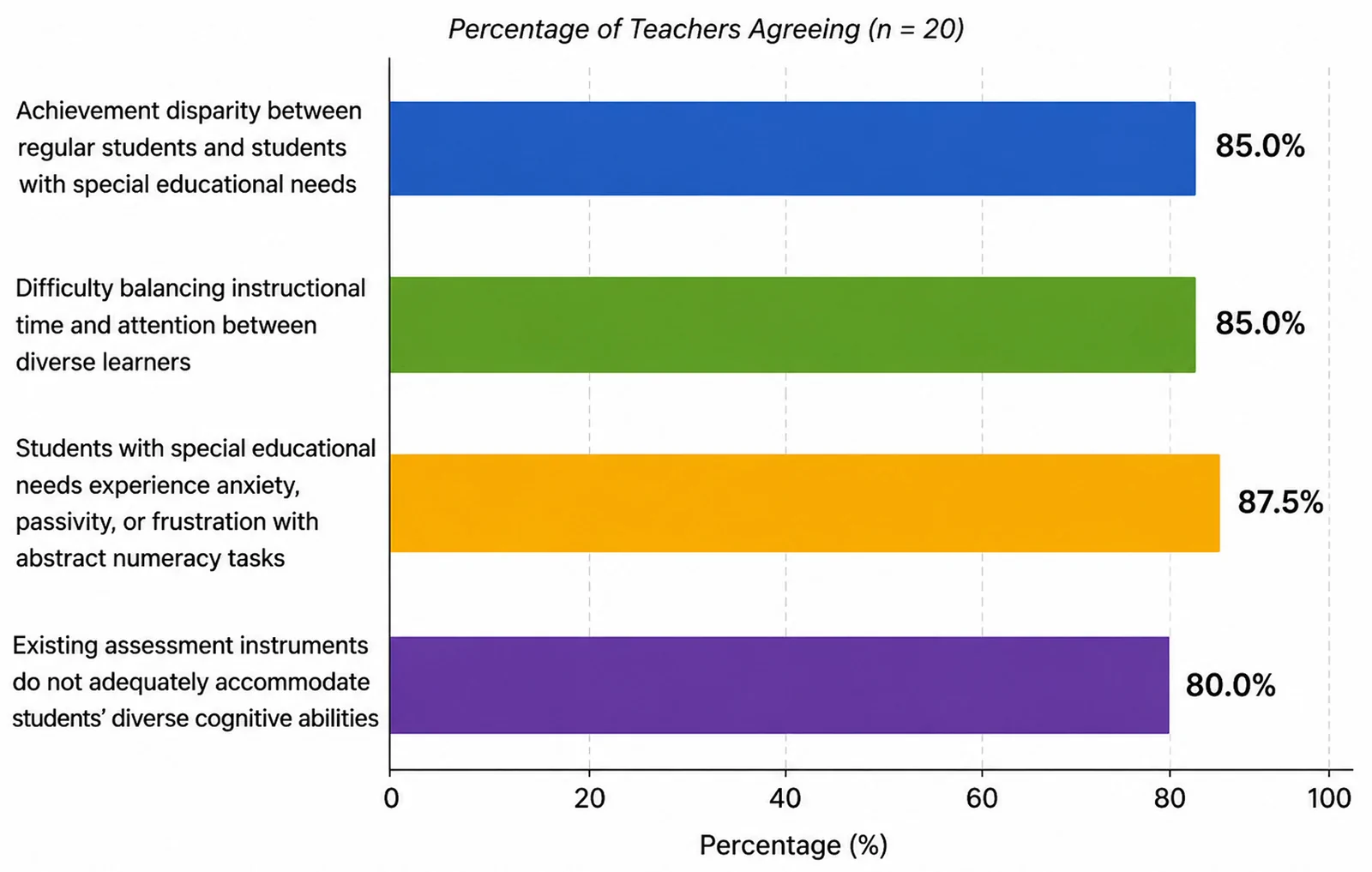
Current conditions of numeracy learning in inclusive elementary classrooms.

The findings indicate that teachers consistently perceived considerable challenges in implementing numeracy instruction within inclusive classrooms. The overall percentage for this dimension reached 84.37%, indicating a high level of agreement regarding the existence of instructional problems. Specifically, 87.50% of teachers agreed that students with special educational needs often experience anxiety, passivity, or frustration when dealing with abstract numeracy tasks. Likewise, 85.00% reported substantial disparities in numeracy achievement between regular students and students with special educational needs, while another 85.00% acknowledged difficulties in balancing instructional support for students with diverse learning characteristics. The lowest percentage within this dimension was related to assessment practices, with 80.00% of teachers indicating that current assessment instruments do not adequately accommodate students’ diverse cognitive abilities. Overall, these findings demonstrate that numeracy learning in inclusive classrooms is characterized by considerable heterogeneity in students’ learning needs, requiring instructional approaches that provide equitable learning opportunities for learners with different levels of readiness and support needs.

### 3.2 Limitations of current problem-based learning

The second component of the needs assessment explored teachers’ perceptions regarding the implementation of conventional Problem-Based Learning (PBL) in inclusive classrooms. Three indicators were examined, and the results are presented in Figure 3.

**Figure 3.**
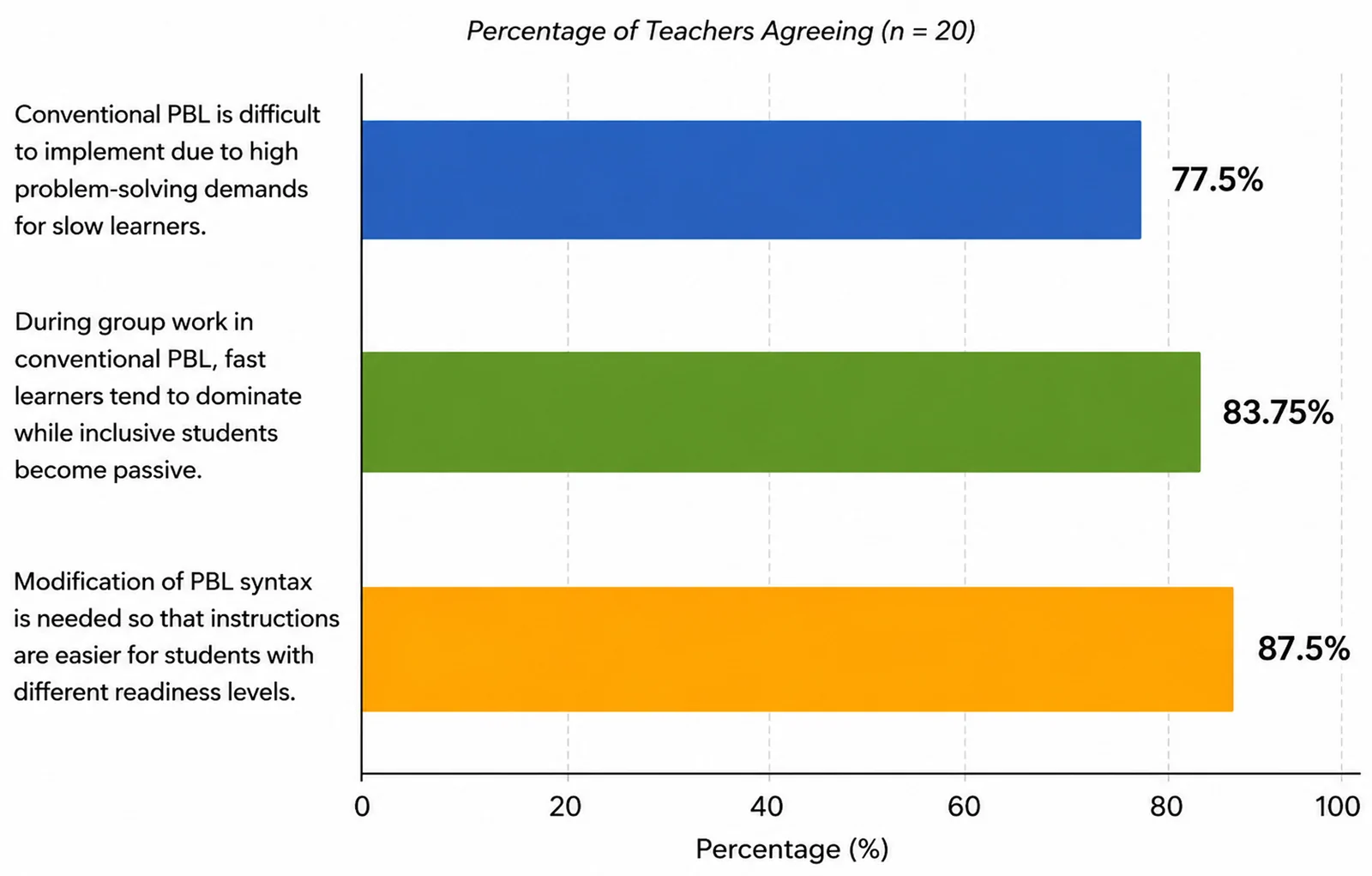
Current implementation of the problem based learning model.

The findings reveal that teachers perceived several limitations in implementing conventional PBL in inclusive settings. Overall, this dimension obtained an average percentage of 82.92%, indicating that teachers generally regarded the current PBL model as insufficiently responsive to heterogeneous classrooms. Among the three indicators, the strongest agreement (87.50%) was related to the need for modifying the existing PBL syntax to accommodate students with different levels of learning readiness. Similarly, 83.75% of teachers reported that fast learners frequently dominate collaborative group activities, limiting meaningful participation by students with special educational needs. Meanwhile, 77.50% agreed that conventional PBL is difficult to implement effectively because the cognitive demands of problem-solving activities are often too high for slow learners.

These findings suggest that although Problem-Based Learning remains a valuable student-centered approach, its current implementation requires systematic adaptation to ensure equitable participation and accessibility for learners with diverse educational needs.

### 3.3 Challenges in Implementing Differentiated Instruction

The third component of the needs assessment investigated teachers’ experiences in implementing differentiated instruction during numeracy learning. The findings are summarized in Figure 4.

**Figure 4.**
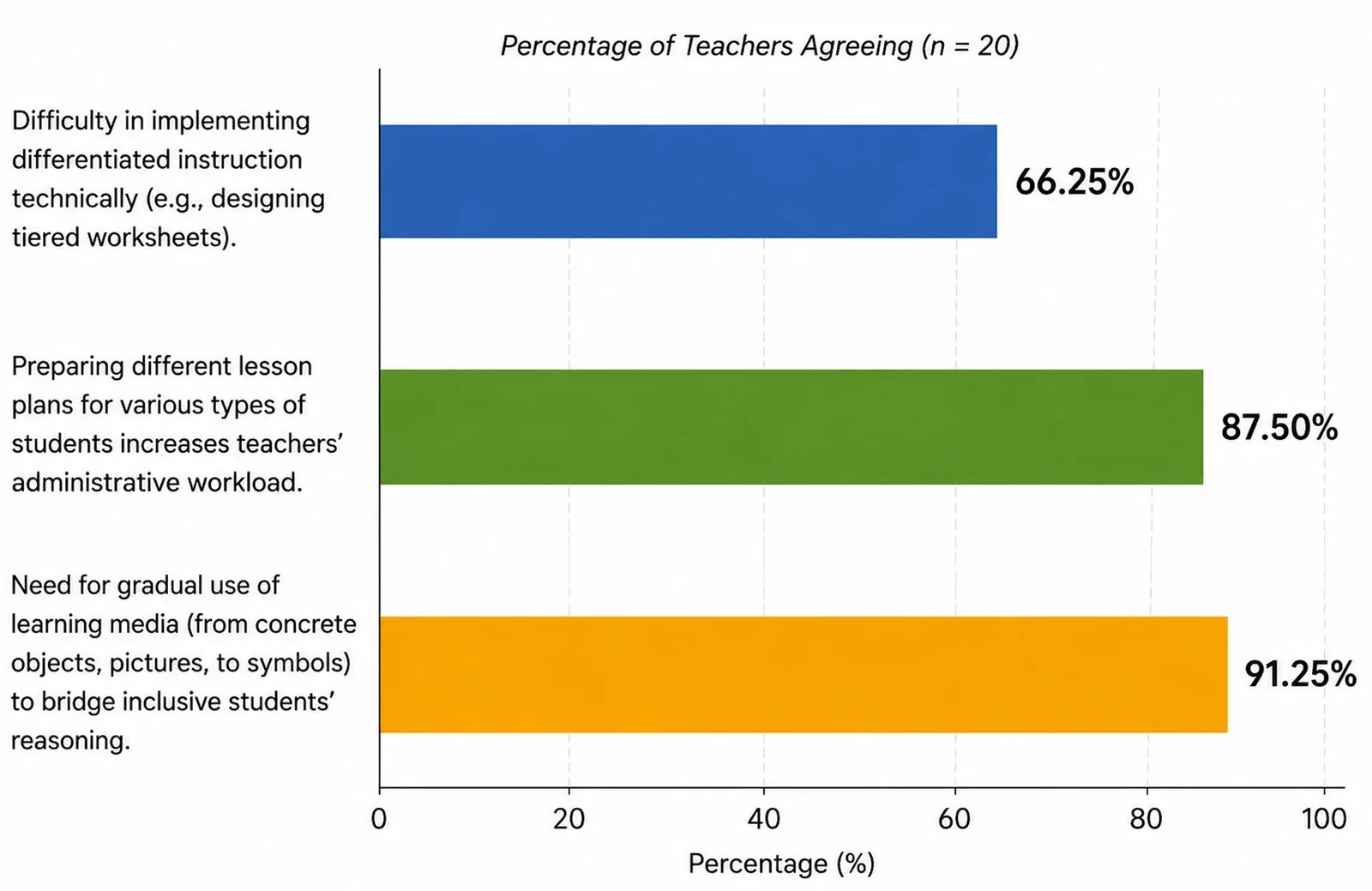
Implementation of the differentiated instruction approach.

Overall, teachers expressed positive attitudes toward differentiated instruction while acknowledging considerable implementation challenges. The average percentage for this dimension was 81.67%. The highest level of agreement (91.25%) concerned the need for gradual learning media progressing from concrete objects to pictorial representations and symbolic forms to support students’ conceptual understanding. Likewise, 87.50% of respondents reported that preparing separate lesson plans and learning materials for students with different characteristics substantially increases teachers’ workload. However, only 66.25% indicated confidence in implementing differentiated instruction technically, suggesting that many teachers continue to experience practical difficulties when designing differentiated worksheets, instructional activities, and classroom management strategies. These findings indicate that teachers recognize the educational value of differentiated instruction but require more practical guidance to implement it systematically in inclusive numeracy classrooms.

### 3.4 Teachers’ urgency for developing the DIF-PBL model

The final component of the questionnaire examined teachers’ perceptions regarding the urgency of developing an instructional model integrating Problem-Based Learning with differentiated instruction. The findings are presented in Figure 5.

**Figure 5.**
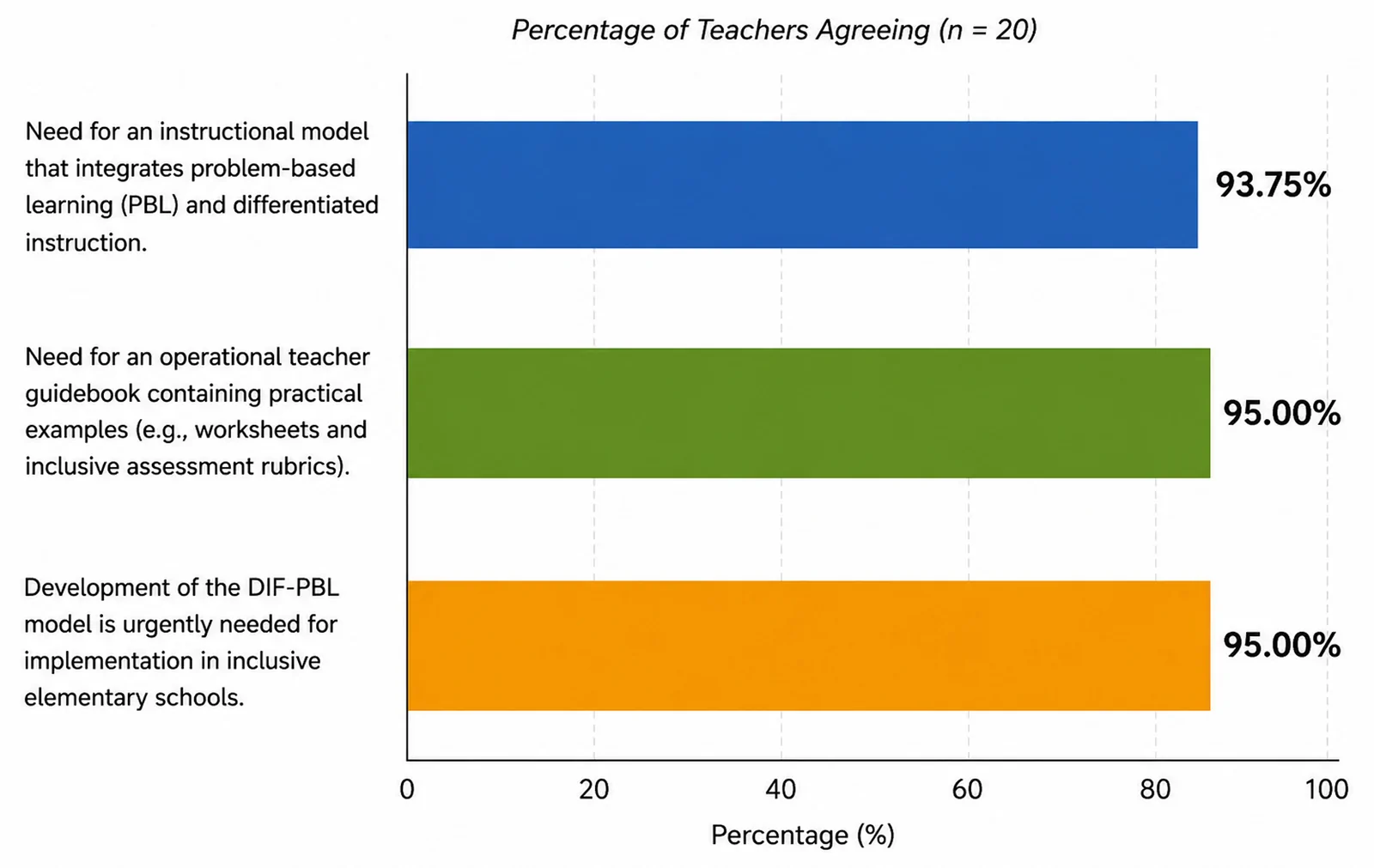
Urgency for developing the DIF-PBL model.

Teachers demonstrated a very high level of consensus regarding the necessity of developing the DIF-PBL model. The overall percentage for this dimension reached 94.58%, representing the highest among all dimensions of the needs assessment. Specifically, 95.00% of respondents agreed that they require an operational teacher guide containing practical examples of lesson plans, differentiated worksheets, and inclusive assessment rubrics. The same percentage (95.00%) agreed that the development of the DIF-PBL model is urgently needed in inclusive elementary schools. Furthermore, 93.75% expressed the need for an instructional model that systematically integrates Problem-Based Learning with differentiated instructional strategies. These findings indicate that teachers perceive current instructional approaches as insufficient for addressing the complexity of inclusive classrooms and strongly support the development of an integrated instructional model that provides practical guidance for implementing inclusive numeracy learning.

### 3.5 Initial student observation

To complement teachers’ perspectives obtained through questionnaires, initial classroom observations were conducted with three students representing different learner profiles: one regular student, one slow learner, and one student with special educational needs (SEN). The observations focused on students’ approaches to solving contextual numeracy tasks, their use of mathematical representations, the level of instructional support required, and their participation during learning activities.

The observations revealed noticeable differences in students’ numeracy performance and learning characteristics. The regular student demonstrated relatively independent problem-solving abilities and was able to move from concrete situations to symbolic mathematical representations with minimal teacher assistance. In contrast, the slow learner required repeated explanations, additional processing time, and continuous guidance before successfully completing contextual numeracy tasks. Meanwhile, the student with special educational needs relied heavily on concrete learning materials, visual representations, and individualized scaffolding to understand mathematical concepts and participate meaningfully in learning activities.

Differences were also observed in classroom participation. While the regular student actively contributed to discussions and collaborative tasks, the slow learner participated only after receiving teacher prompts, whereas the student with special educational needs tended to become passive when learning activities involved abstract mathematical reasoning without sufficient instructional support. These observations reinforce teachers’ perceptions that conventional instructional approaches are insufficient to accommodate the wide range of learner characteristics found in inclusive classrooms.

Overall, the initial student observations support the questionnaire and classroom observation findings by demonstrating that differences in learning readiness, conceptual understanding, and instructional support needs require more adaptive instructional strategies. These findings provide additional empirical evidence for the proposed instructional design requirements of the DIF-PBL model, particularly the incorporation of differentiated learning pathways, Concrete–Pictorial–Abstract (CPA) learning sequences, systematic scaffolding, and flexible instructional support.

### 3.6 Synthesis of the needs assessment

To integrate findings from the questionnaire and initial student observations, a synthesis matrix was developed to identify the major instructional needs, empirical evidence, underlying causes, and corresponding instructional design implications. The synthesis is presented in Table 1.

**Table 1.** Synthesis matrix of the needs analysis for developing the DIF-PBL model.

Needs Assessment Indicator	Key Findings	Critical Root Cause	Instructional Implications for the DIF-PBL Model
Disparities in numeracy achievement between regular students and students with special educational needs	Teachers consistently reported substantial disparities in numeracy achievement between regular students and students with special educational needs.	Existing numeracy instruction continues to rely on relatively uniform learning activities despite considerable variation in students’ readiness and learning characteristics.	Develop differentiated learning pathways that accommodate learner diversity while maintaining common instructional objectives.
Difficulty balancing instructional support among diverse learners	Teachers experienced considerable difficulty in balancing instructional time and support across students with different learning characteristics.	Teachers are required to simultaneously address diverse learning needs within the same classroom using limited adaptive instructional strategies.	Integrate flexible teacher support, adaptive classroom management, and differentiated instructional pathways within the DIF-PBL model.
Students with special educational needs experience anxiety when learning abstract numeracy	Students with special educational needs frequently demonstrate anxiety, passivity, and conceptual difficulties when learning abstract mathematical concepts.	Instruction often progresses directly toward symbolic representation without sufficient conceptual support.	Integrate the Concrete–Pictorial–Abstract (CPA) learning sequence together with systematic scaffolding.
Existing assessment practices do not adequately accommodate learner diversity	Current assessment practices remain insufficiently responsive to students’ diverse cognitive abilities.	Assessment predominantly applies uniform criteria to heterogeneous learners.	Develop adaptive formative assessments and differentiated assessment strategies.
Conventional Problem-Based Learning is difficult for slow learners	Teachers perceived conventional PBL as placing excessive cognitive demands on slow learners.	Existing PBL procedures assume relatively homogeneous learner readiness and provide limited instructional flexibility.	Differentiate task complexity and provide graduated instructional support throughout the PBL process.
Fast learners dominate collaborative learning activities	Collaborative learning frequently results in unequal participation, with fast learners dominating group activities while students requiring greater support become passive.	Collaborative learning lacks structured mechanisms that ensure equitable participation.	Incorporate structured peer tutoring, collaborative roles, and balanced participation strategies.
Existing PBL syntax requires modification	Teachers considered the current PBL syntax insufficiently flexible to accommodate students with different levels of learning readiness.	Conventional PBL provides limited opportunities for adaptive instruction.	Develop flexible and adaptive DIF-PBL syntax that supports differentiated learning pathways.
Teachers experience difficulty implementing differentiated instruction	Teachers recognized the value of differentiated instruction but experienced practical difficulties implementing it consistently.	Limited practical guidance for designing differentiated learning activities and instructional materials.	Provide operational guidance, practical examples, and differentiated instructional resources within the DIF-PBL model.
Preparing differentiated lesson plans increases teachers’ workload	Teachers perceived differentiated lesson planning as increasing administrative workload.	Differentiation often requires multiple instructional resources and lesson plans.	Develop integrated lesson-planning templates that support differentiation while reducing teachers’ workload.
Students require gradual learning progression	Teachers emphasized the importance of progressing from concrete experiences toward pictorial and symbolic mathematical representations.	Students require structured conceptual progression before engaging with abstract mathematical ideas.	Embed CPA learning sequences consistently throughout all instructional phases of the DIF-PBL model.
Teachers need an integrated instructional model	Teachers expressed the need for an instructional model integrating Problem-Based Learning with Differentiated Instruction.	Existing instructional approaches address PBL and differentiation separately rather than as a unified framework.	Develop an integrated DIF-PBL instructional model combining inquiry-based learning with differentiated instruction.
Teachers require an operational implementation guide	Teachers emphasized the importance of practical guidance containing lesson examples, differentiated worksheets, and inclusive assessment rubrics.	Teachers require operational resources to implement inclusive numeracy instruction effectively.	Produce a comprehensive teacher guidebook supporting classroom implementation of the DIF-PBL model.
Development of the DIF-PBL model is urgently needed	Teachers demonstrated a strong consensus regarding the urgency of developing an instructional model specifically designed for inclusive numeracy learning.	Existing instructional approaches remain insufficient to address the complexity of inclusive classrooms.	Develop and validate the DIF-PBL model as an evidence-based instructional innovation for inclusive elementary education.

The synthesis demonstrates that the instructional challenges identified by teachers are highly interconnected rather than isolated classroom issues. The observed disparities in students’ numeracy achievement, teachers’ difficulties in managing heterogeneous classrooms, mathematics anxiety among students with special educational needs, and limitations of current assessment practices collectively indicate that existing instructional approaches remain insufficiently responsive to learner diversity. Similarly, limitations identified in the implementation of conventional Problem-Based Learning—including excessive cognitive demands, unequal participation during collaborative learning, and inflexible instructional procedures—further reinforce the need for systematic instructional adaptation.

The findings also reveal that while differentiated instruction offers considerable potential for accommodating diverse learning needs, teachers require practical guidance to implement differentiation effectively. Difficulties in preparing differentiated instructional materials, designing adaptive assessments, and managing multiple learning pathways indicate that differentiation cannot simply be added to existing classroom practices but should instead be embedded within a coherent instructional framework.

Taken together, these findings suggest that the development of the DIF-PBL model should address four major instructional design requirements. First, the model should incorporate adaptive learning pathways that accommodate students’ diverse readiness levels and learning profiles. Second, it should modify conventional PBL syntax to ensure equitable participation among heterogeneous learners. Third, the model should integrate gradual learning experiences supported by concrete, pictorial, and abstract representations together with systematic scaffolding strategies. Finally, the instructional framework should provide teachers with practical operational guidance, including differentiated learning activities, adaptive assessment procedures, and classroom management strategies that are feasible for implementation in inclusive elementary classrooms. These instructional design requirements constitute the empirical foundation for the subsequent development of the Differentiation-Based Problem-Based Learning (DIF-PBL) model.

## 4. Discussion

### 4.1 Why inclusive numeracy learning requires adaptive instruction

The findings of this study indicate that numeracy learning in inclusive elementary classrooms is characterized by substantial heterogeneity in students’ learning readiness, mathematical understanding, and instructional support needs. Teachers consistently reported difficulties in simultaneously facilitating students with diverse cognitive abilities while ensuring equitable participation in classroom learning. These findings reinforce the fundamental principle of inclusive education that instructional practices should be designed to accommodate learner diversity rather than expecting students to adapt to uniform teaching approaches ( Dr. Ranbir, 2024; Kaur & Bhatia, 2024; Letzel-Alt & Pozas, 2025).

From a cognitive development perspective, Piaget’s theory suggests that elementary school students are predominantly situated within the concrete operational stage, during which mathematical understanding develops most effectively through direct manipulation of concrete objects before progressing toward symbolic abstraction ( Wadsworth, 1995). However, students with special educational needs frequently require extended experiences with concrete representations and additional instructional support before achieving conceptual understanding comparable to their peers ( Flores et al., 2023; Flores & Hinton, 2022). Consequently, identical instructional activities may produce substantially different learning outcomes among students within the same classroom.

The present findings are also consistent with Tomlinson’s theory of Differentiated Instruction, which emphasizes that effective teaching should be responsive to students’ readiness, interests, and learning profiles ( Economidou Stavrou, 2024; Tomlinson, 2021). Teachers participating in this study recognized the necessity of adapting learning activities according to students’ diverse characteristics, yet they simultaneously acknowledged experiencing considerable challenges in implementing differentiation consistently. This discrepancy suggests that awareness of differentiation alone is insufficient; teachers require structured instructional frameworks that operationalize differentiation within daily classroom practice.

Furthermore, the findings support the principles of Universal Design for Learning (UDL), which advocate providing multiple means of engagement, representation, and action or expression to ensure equitable learning opportunities for all students. The reported disparities in numeracy achievement and the limitations of existing assessment practices indicate that inclusive numeracy learning requires instructional environments capable of accommodating diverse pathways toward achieving common learning objectives. Therefore, adaptive instruction should not be viewed as an optional teaching strategy but rather as a fundamental requirement for achieving educational equity in inclusive classrooms.

### 4.2 Why conventional problem-based learning falls short in inclusive classrooms

Although Problem-Based Learning has consistently demonstrated positive effects on students’ critical thinking ( Nguyen et al., 2025), collaboration ( Wijayanto et al., 2025), and mathematical problem-solving abilities ( Dorimana et al., 2022), the present findings reveal several limitations when conventional PBL is implemented within inclusive classrooms. Teachers reported that existing PBL procedures are insufficiently flexible to accommodate learners with diverse cognitive readiness and frequently result in unequal participation during collaborative learning activities.

One possible explanation can be understood through Cognitive Load Theory. Authentic contextual problems, while educationally meaningful, often require learners to simultaneously process multiple sources of information, activate prior knowledge, coordinate collaborative interactions, and construct appropriate solution strategies ( Evains et al., 2024; Lovellette et al., 2024). For students with limited working memory capacity or delayed cognitive development, these instructional demands may generate excessive cognitive load, thereby reducing learning effectiveness. Consequently, the complexity of conventional PBL tasks may unintentionally disadvantage students with special educational needs unless appropriate instructional supports are incorporated ( D’Elia et al., 2025; Riera Hermida et al., 2025).

The collaborative nature of PBL also presents unique challenges within heterogeneous classrooms. Collaborative learning theories emphasize that group interaction promotes knowledge construction through social negotiation and shared responsibility ( Hmelo-Silver & Barrows, 2008; Zabolotna et al., 2025). However, the findings indicate that fast learners frequently dominate discussions and decision-making processes, while students requiring greater instructional support become passive participants. Such unequal participation limits opportunities for meaningful engagement and may reinforce existing achievement disparities rather than reducing them.

These findings do not suggest that Problem-Based Learning is inappropriate for inclusive education. Instead, they indicate that the instructional syntax of conventional PBL requires systematic adaptation to ensure that inquiry, collaboration, and problem-solving activities remain accessible for students with varying learning characteristics. Consequently, the effectiveness of PBL within inclusive classrooms depends not only on authentic problems but also on the flexibility of instructional design supporting diverse learners throughout the learning process.

### 4.3 Instructional design requirements emerging from the needs assessment

Beyond identifying instructional challenges, the present study provides empirical evidence regarding the instructional features required to support effective numeracy learning in inclusive classrooms. The synthesis of questionnaire findings and student observations indicates that several instructional components consistently emerged as essential design requirements for the proposed DIF-PBL model.

First, the need for Concrete–Pictorial–Abstract (CPA) learning sequences emerged from teachers’ observations that many students, particularly those with special educational needs, experienced anxiety and conceptual difficulties when learning abstract mathematical concepts. The CPA approach enables students to construct mathematical understanding gradually by progressing from concrete manipulation to visual representation before introducing symbolic abstraction, thereby reducing conceptual barriers during learning.

Second, the findings highlight the necessity of tiered learning tasks. Teachers reported considerable variation in students’ readiness levels, suggesting that identical learning activities cannot adequately address the needs of all learners. Tiered tasks provide differentiated levels of complexity while maintaining common instructional objectives, enabling students to engage with meaningful mathematical problems according to their current developmental readiness.

Third, systematic scaffolding emerged as another critical instructional requirement. Students requiring additional learning support often demonstrated improved participation when teachers provided structured guidance, prompting questions, worked examples, or step-by-step assistance during mathematical problem solving. These findings align with sociocultural perspectives emphasizing the importance of instructional support within students’ zones of proximal development.

Fourth, the findings suggest that peer tutoring should become an integral component of collaborative learning within inclusive classrooms. Rather than allowing naturally occurring dominance by high-achieving students, structured peer tutoring creates reciprocal learning relationships in which students provide purposeful academic assistance while maintaining active participation by all group members. Such arrangements have the potential to strengthen both academic achievement and social inclusion.

Finally, teachers consistently emphasized the need for a more flexible instructional syntax capable of integrating differentiated learning activities within Problem-Based Learning. Rather than following a fixed sequence of instructional procedures, the proposed model should incorporate adaptive decision points that enable teachers to modify learning pathways according to students’ readiness, learning profiles, and instructional progress. Collectively, these instructional requirements represent practical design principles derived directly from empirical classroom evidence rather than theoretical assumptions alone.

### 4.4 Toward the development of the DIF-PBL model

The present study constitutes the empirical foundation for developing the Differentiation-Based Problem-Based Learning (DIF-PBL) model. Rather than combining Problem-Based Learning and Differentiated Instruction conceptually, the needs assessment demonstrates how specific classroom challenges can be translated into concrete instructional design principles.

The proposed DIF-PBL model is therefore expected to integrate several complementary instructional components. Problem-Based Learning will continue to function as the primary pedagogical framework for promoting inquiry, authentic problem solving, and collaborative learning. Differentiated Instruction will provide adaptive mechanisms for accommodating students’ readiness, interests, and learning profiles throughout each stage of instruction. The Concrete–Pictorial–Abstract approach will support conceptual development by reducing abstraction during numeracy learning, while scaffolding and peer tutoring will facilitate equitable participation among heterogeneous learners. Flexible instructional syntax and adaptive assessment strategies will enable teachers to implement differentiation systematically without substantially increasing instructional complexity.

Accordingly, the findings of this study extend beyond identifying teachers’ instructional needs. They provide an empirically grounded set of instructional design principles that will inform the subsequent stages of model development, including prototype construction, expert validation, field implementation, and effectiveness evaluation. These stages will be reported in subsequent publications.

Figure 6 illustrates how the findings of the needs assessment were systematically translated into instructional design principles. Rather than functioning solely as descriptive findings, the identified instructional problems became the basis for determining adaptive instructional components, including differentiated learning pathways, CPA sequencing, scaffolding, tiered tasks, peer tutoring, and flexible Problem-Based Learning syntax. Consequently, the DIF-PBL model was conceptualized as an evidence-informed instructional framework grounded in authentic classroom needs rather than theoretical assumptions alone.

**Figure 6.**
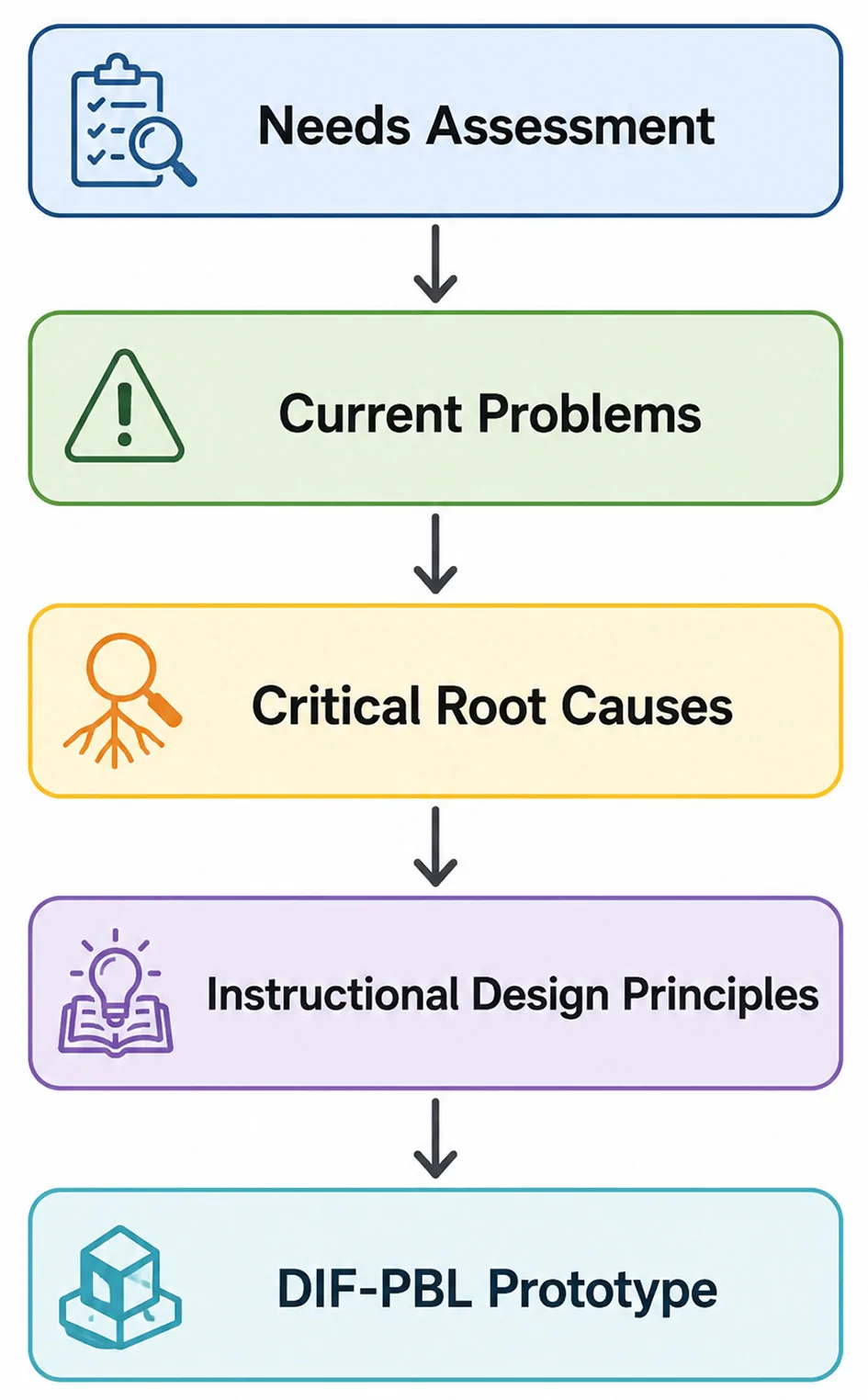
Translation of Needs Assessment into the DIF-PBL Prototype.

Unlike previous studies that have independently investigated Problem-Based Learning or Differentiated Instruction, this study establishes an empirical design pathway linking teachers’ instructional needs, root cause analysis, and instructional design principles for inclusive numeracy learning. The proposed DIF-PBL model is therefore not merely a conceptual integration of two pedagogical approaches but an evidence-informed instructional framework derived systematically from authentic classroom needs. This empirical design orientation represents the primary novelty of the present study and provides a stronger foundation for subsequent model development and validation.

### 4.5 Research contributions

This study contributes to the literature in three important ways. First, it enriches research on inclusive mathematics education by providing a comprehensive empirical analysis of teachers’ instructional needs in numeracy learning, an area that has received relatively limited attention compared with studies evaluating instructional effectiveness. Second, the study bridges the gap between needs assessment and instructional design by demonstrating how empirical classroom evidence can be systematically translated into design requirements for developing an adaptive learning model. This design-oriented perspective extends previous studies that primarily described instructional challenges without generating explicit instructional implications.

Third, the study introduces an evidence-based conceptual foundation for the development of the Differentiation-Based Problem-Based Learning (DIF-PBL) model. Rather than proposing an instructional innovation solely from theoretical considerations, the proposed model is grounded in teachers’ experiences, classroom observations, and students’ learning characteristics. This approach strengthens the theoretical and practical relevance of the subsequent model development process and provides a replicable framework for future instructional design research in inclusive education.

### 4.6 Limitations and future research

Several limitations should be acknowledged. First, the study was conducted in a limited number of inclusive elementary schools within one regency, which may restrict the generalizability of the findings to other educational contexts. Second, the needs assessment primarily reflects teachers’ perspectives, while direct evidence from students and parents remains limited. Third, the present study represents only the initial stage of a broader Research and Development project and therefore does not evaluate the effectiveness of the proposed instructional model.

Future research should focus on developing and validating the DIF-PBL prototype based on the instructional design requirements identified in this study. Subsequent investigations should include expert validation, classroom implementation across diverse inclusive settings, and rigorous evaluation of the model’s effectiveness in improving students’ numeracy achievement, conceptual understanding, classroom participation, and learning equity. In addition, future studies may explore the integration of digital learning technologies and adaptive assessment systems to further enhance differentiated numeracy instruction within inclusive elementary education.

## Conclusion

This study conducted a comprehensive needs assessment as the initial phase of developing a Differentiation-Based Problem-Based Learning (DIF-PBL) model for numeracy learning in inclusive elementary schools. The findings revealed that current numeracy instruction faces significant challenges arising from the heterogeneity of students’ learning characteristics, limitations of conventional Problem-Based Learning implementation, and practical difficulties encountered by teachers in applying differentiated instruction. Teachers consistently reported that existing instructional approaches do not sufficiently accommodate differences in students’ readiness, learning profiles, and support needs, thereby limiting equitable participation and meaningful learning experiences in inclusive classrooms.

The integrated analysis of questionnaire data and student observations identified several essential instructional design requirements for the proposed DIF-PBL model. These include adaptive learning pathways, modified Problem-Based Learning syntax, Concrete–Pictorial–Abstract (CPA) learning sequences, systematic scaffolding, structured peer tutoring, tiered learning tasks, and adaptive assessment strategies. Collectively, these findings demonstrate that effective numeracy instruction in inclusive classrooms requires more than the adoption of individual instructional strategies; it requires a coherent instructional framework that systematically integrates learner diversity into every stage of the learning process.

The primary contribution of this study lies in bridging the gap between empirical needs assessment and instructional design. Rather than merely identifying instructional problems, the study translates classroom evidence into a set of evidence-based instructional design principles that provide the conceptual foundation for the development of the DIF-PBL model. This contribution extends previous studies that have examined Problem-Based Learning and Differentiated Instruction separately by offering an integrated framework specifically intended for inclusive numeracy education.

Future research should focus on the subsequent stages of the Research and Development process, including prototype development, expert validation, classroom implementation, and effectiveness evaluation across diverse inclusive school settings. Further investigations are also needed to examine the long-term impact of the DIF-PBL model on students’ numeracy achievement, conceptual understanding, learning engagement, and educational equity, as well as to explore the integration of digital technologies and adaptive learning systems to strengthen differentiated numeracy instruction.

## Ethical approval

This study was conducted in accordance with the principles of the Declaration of Helsinki. Ethical approval was obtained from the Institutional Review Board (IRB) of Universitas Bosowa (Approval No. 203/1.02/DRIPM-UNIBOS/VII/2025; approved on 21 July 2025).

## AI statement

The authors stated that generative artificial intelligence tools were used in a limited manner to assist with language editing, organization of academic writing, improvement of clarity, and improving image quality. The conceptual framework, research design, data analysis, interpretation, and final conclusions were developed entirely by the authors.

## Informed consent

Written informed consent was obtained from all participating teachers. For student participants who were minors, written informed consent was obtained from their parents or legal guardians prior to participation. Assent was also obtained from the students according to their level of understanding.

## Data Availability

The data supporting the findings of this study are available in figshare:
• Teacher Needs Analysis Instrument. DOI: https://doi.org/10.6084/m9.figshare.33092594 ( Putri, 2026a) This repository contains the teacher needs analysis questionnaire, anonymized raw responses from 20 participating teachers, and the descriptive data used to generate the quantitative findings reported in this study.
• Structured Classroom Observation Sheet. DOI: https://doi.org/10.6084/m9.figshare.33130376 ( Putri, 2026b). This repository contains the structured classroom observation instrument together with anonymized observation summaries for the three learner profiles (regular student, slow learner, and student with special educational needs) used during the initial needs assessment. All datasets have been anonymized to protect participant confidentiality. Data are available under the terms of the Creative Commons Attribution 4.0 International (CC-BY 4.0) license.
