# From Needs Assessment to Instructional Design: Empirical Requirements for a DIF-PBL Model in Inclusive Numeracy Learning

**DOI:** 10.12688/f1000research.187953.2

**Published:** 2026-11-04

**Authors:** Mentari Rama Putri, Arismunandar Arismunandar, Syafruddin Side, Erma Suryani Sahabuddin, Ahmad Swandi

**Affiliations:** 1 Doctoral Program in Educational Sciences, Postgraduate Program,, State University of Makassar, Makassar, South Sulawesi, Indonesia; 2 Educational Administration Study Program, State University of Makassar, Makassar, South Sulawesi, Indonesia; 3 Mathematics Study Program, State University of Makassar, Makassar, South Sulawesi, Indonesia; 4 Population & Environmental Education Study Program, State University of Makassar, Makassar, South Sulawesi, Indonesia; 5 Science Education Study Program, Bosowa University, Makassar, South Sulawesi, Indonesia

**Keywords:** inclusive education; numeracy learning; problem-based learning; differentiated instruction; needs assessment; instructional design.

## Abstract

Inclusive elementary classrooms require instructional approaches that accommodate substantial variation in students’ numeracy readiness, learning characteristics, and support needs. This study examined teachers’ instructional needs and preliminary learner differences as an empirical basis for designing a Differentiation-Based Problem-Based Learning (DIF-PBL) model for inclusive numeracy learning. The study was conducted as a descriptive needs assessment within a broader research and development project. Participants included 20 classroom teachers and special education support teachers from three inclusive elementary schools in Bone Regency, South Sulawesi, Indonesia. Data were collected using a 13-item four-point Likert questionnaire and structured observations of three students representing different learner profiles. Questionnaire data were analyzed using item-level means, standard deviations, and response frequencies, while student observations were treated as illustrative contextual cases. Teachers reported substantial instructional challenges, including disparities in numeracy achievement, difficulty balancing instructional support, limitations in conventional Problem-Based Learning, and practical difficulties in implementing differentiated instruction. The strongest responses concerned the need for an integrated PBL and differentiation model (M = 3.95, SD = 0.22), an operational teacher guide (M = 4.00, SD = 0.00), and the urgency of developing the DIF-PBL model (M = 4.00, SD = 0.00). The findings support several design requirements, including differentiated learning pathways, flexible PBL procedures, Concrete–Pictorial–Abstract learning progression, systematic scaffolding, adaptive assessment, and practical teacher guidance. The findings provide an empirical starting point for subsequent prototype development and validation rather than evidence of model effectiveness.

## 1. Introduction

Numeracy has become one of the most essential competencies required for individuals to successfully participate in twenty-first-century society ( Rosnelli & Ristiana, 2023). Beyond the ability to perform arithmetic operations, numeracy encompasses the capacity to interpret quantitative information, apply mathematical reasoning, solve contextual problems, and make evidence-based decisions in everyday life ( Geiger & Schmid, 2024). These competencies are increasingly important in an era characterized by rapid technological advancement, data-driven decision-making, and complex societal challenges. Consequently, numeracy is now recognized as a fundamental component of educational quality and a key determinant of students’ future academic achievement, employability, and lifelong learning.

The growing importance of numeracy has been consistently emphasized through international educational assessments. The Programme for International Student Assessment (PISA) defines mathematical literacy as an individual’s ability to formulate, employ, and interpret mathematics in various real-world contexts. Rather than merely measuring procedural calculation, PISA evaluates students’ capacity to reason mathematically, model authentic situations, and solve unfamiliar problems ( OECD, 2022). Despite continuous educational reforms, Indonesian students continue to demonstrate relatively low levels of mathematical literacy ( OECD, 2022). The 2022 PISA results showed that Indonesia’s mathematics performance remained substantially below the OECD average, indicating that many students still struggle to transfer mathematical concepts into meaningful real-life applications. Similar concerns are reflected in the Indonesian Minimum Competency Assessment (Asesmen Kompetensi Minimum/AKM), which identifies numeracy as one of the two core competencies expected of all learners ( Kemendikbudristek, 2021). National education reports consistently indicate that a considerable proportion of elementary school students have not yet achieved the expected level of numeracy proficiency, highlighting the urgent need for instructional innovations capable of improving meaningful mathematical understanding ( Yeh et al., 2019).

These challenges become considerably more complex within inclusive elementary schools. Inclusive education requires students with diverse academic abilities, learning characteristics, and developmental needs to learn together within the same classroom environment ( Moriña, 2017; Shaeffer, 2019). In a typical inclusive classroom, teachers simultaneously accommodate regular students, fast learners, slow learners, and students with special educational needs, each possessing distinct cognitive readiness, learning profiles, communication abilities, and support requirements ( De leon et al., 2024; Jardinez & Natividad, 2024). Such heterogeneity creates substantial instructional challenges because teachers must ensure equitable learning opportunities while maintaining curriculum standards for all students ( Lotan, 2006). Consequently, instructional decisions can no longer rely on uniform teaching strategies designed for an “average” learner but must instead accommodate substantial variations in students’ readiness and learning needs.

Numeracy instruction presents particularly significant difficulties in inclusive classrooms because mathematical concepts often progress from concrete experiences toward increasingly abstract representations ( Abdulah et al., 2025; Nair et al., 2024). Students with special educational needs frequently require extended scaffolding, repeated learning experiences, concrete instructional media, and individualized guidance before they are able to understand symbolic mathematical representations ( Evains et al., 2024; Mutodi & Mosimege, 2021; Van Garderen et al., 2018). In contrast, regular students and fast learners generally progress more rapidly through instructional activities. This discrepancy often creates instructional dilemmas in which teachers struggle to divide their attention effectively across students with markedly different learning characteristics ( Goyibova et al., 2025; Smets et al., 2022). As a result, students with special educational needs may experience mathematics anxiety, reduced classroom participation, and persistent learning difficulties, whereas higher-achieving students dominate classroom discussions and collaborative activities. Such conditions frequently widen the achievement gap rather than promoting equitable learning outcomes.

Problem-Based Learning (PBL) has long been recognized as one of the most effective student-centered instructional models for developing higher-order thinking skills, problem-solving ability, collaboration, and conceptual understanding ( Astawan et al., 2025; Suryawati et al., 2020). Through authentic contextual problems, students actively construct knowledge while engaging in inquiry, discussion, and collaborative investigation ( Chin & Chia, 2004; Martawijaya et al., 2023). Numerous studies have demonstrated the positive contribution of PBL to mathematics learning and numeracy development ( Ssali et al., 2025; Suparman et al., 2021). Consequently, PBL has been widely recommended as an instructional approach capable of fostering meaningful learning experiences that extend beyond procedural knowledge.

However, implementing conventional PBL within inclusive classrooms remains challenging. The characteristics that make PBL effective in general classrooms may also create barriers for students with diverse learning needs. Complex problem scenarios often impose high cognitive demands that exceed the learning readiness of slow learners and students with special educational needs. Open-ended investigations frequently require students to independently regulate learning processes, interpret lengthy contextual information, and participate actively in collaborative discussions ( Emara et al., 2021; Kinnebrew et al., 2017). In heterogeneous groups, fast learners commonly dominate decision-making and task completion, whereas students with special educational needs become passive observers with limited opportunities to contribute meaningfully ( Theobald et al., 2017; Zeng et al., 2024). Furthermore, conventional PBL generally may provide limited flexibility for adapting learning activities, assessment strategies, and teacher support to substantial differences in learner readiness and therefore provides limited instructional flexibility for adapting learning activities, assessment strategies, and teacher support according to individual learning differences. These limitations suggest that although PBL possesses substantial pedagogical potential, its implementation within inclusive classrooms requires systematic instructional adaptation.

Differentiated Instruction has emerged as one of the most promising approaches for addressing learner diversity within inclusive education ( Goyibova et al., 2025; Nketsia et al., 2025). Grounded in the work of Tomlinson (2021), differentiated instruction emphasizes that effective teaching should be responsive to students’ readiness, interests, and learning profiles ( Coubergs et al., 2017; Gheyssens et al., 2022). Rather than providing identical learning experiences for every student, teachers systematically modify instructional content, learning processes, expected products, and classroom environments to ensure that all learners receive appropriate levels of challenge and support. Within numeracy instruction, differentiated teaching enables students to progress according to their individual developmental stages while maintaining common learning objectives.

Despite its considerable potential, differentiated instruction is often difficult to implement consistently in everyday classroom practice. Many teachers experience uncertainty when designing differentiated learning materials, preparing tiered instructional activities, developing adaptive assessments, and managing multiple learning pathways simultaneously. Preparing different lesson plans and instructional resources for diverse student groups substantially increases teachers’ workload and administrative responsibilities. Consequently, differentiation is frequently implemented only partially and remains disconnected from existing instructional models. In particular, previous implementations of differentiated instruction have rarely been systematically integrated with the inquiry-oriented learning processes characteristic of Problem-Based Learning, leaving teachers without a coherent instructional framework specifically designed for inclusive numeracy education.

Existing studies have provided evidence regarding the potential of Problem-Based Learning and Differentiated Instruction to support learning, but these approaches have often been examined separately or evaluated after implementation. Less attention has been given to how teachers’ actual instructional needs in inclusive numeracy classrooms can be systematically translated into concrete instructional requirements for an integrated model. In particular, there is limited empirical evidence showing how differences in learners’ numeracy readiness, difficulties in implementing conventional PBL, challenges in applying differentiated instruction, and teachers’ need for practical instructional support can be connected to specific design decisions within an integrated instructional framework.

Addressing this gap requires a needs assessment that goes beyond establishing whether teachers perceive instructional problems. It should identify the specific classroom conditions and instructional challenges that need to be addressed and establish a traceable relationship between empirical findings, instructional requirements, and preliminary design implications. Such an approach can help ensure that an instructional model is grounded in classroom realities rather than being derived primarily from theoretical assumptions.

Therefore, the present study focused on the needs-assessment phase of a broader Research and Development project for the Differentiation-Based Problem-Based Learning (DIF-PBL) model. The study examined teachers’ perceptions of current numeracy learning conditions, the implementation of conventional PBL, challenges in implementing differentiated instruction, and the perceived need for an integrated instructional approach. These findings were subsequently mapped to preliminary instructional requirements and design implications for the DIF-PBL model. The study did not evaluate the effectiveness of DIF-PBL; rather, it established an empirical basis for its subsequent development and validation. Accordingly, this study addressed the following research questions:

RQ1. What are the current conditions of numeracy learning in inclusive elementary classrooms?

RQ2. What limitations do teachers experience in implementing conventional Problem-Based Learning?

RQ3. What challenges do teachers encounter in implementing differentiated instruction?

RQ4. What preliminary instructional requirements can be derived from the needs assessment to inform the design of the DIF-PBL model?

## 2. Research methods

### 2.1 Research design

This study represents theneeds-assessment phase of a broader research and development project aimed at designing a Differentiation-Based Problem-Based Learning (DIF-PBL) model for inclusive numeracy learning. Rather than evaluating the effectiveness of the model, the present study focused on identifying instructional problems, teachers’ perceived needs, and preliminary differences in learner characteristics that could inform subsequent instructional design.

A descriptive needs-assessment design was employed. The primary dataset consisted of a structured questionnaire administered to teachers, while structured observations of three students were used as illustrative contextual evidence. The questionnaire provided quantitative information about teachers’ perceptions of current numeracy instruction, conventional Problem-Based Learning, differentiated instruction, and the perceived need for an integrated instructional model. The student observations were not intended to provide population-level evidence but to illustrate differences in learning readiness, mathematical representation, instructional support needs, and classroom participation.

The findings from these two sources were subsequently synthesized to formulate instructional design requirements for the DIF-PBL model. Because the present study did not collect or report qualitative teacher interviews, focus groups, or open-ended questionnaire data, it is not characterized as a convergent parallel mixed-methods study.

### 2.2 Participants

This research was conducted from August 2025 to November 2025 in three inclusive elementary schools in Tellu Siattinge District, Bone Regency South Sulawesi, Indonesia. The schools were purposively selected because they formally implemented inclusive education and provided learning opportunities for regular students and students with special educational needs within the same classroom setting. The dissertation research documentation identifies the three schools as SD Negeri 66 Sijelling, SD Negeri 69 Itterung, and SD Negeri 56 Lanca.

Twenty teachers participated in the needs-assessment questionnaire. The participants consisted of classroom teachers and special education support teachers (Guru Pendamping Khusus/GPK) who were involved in inclusive elementary education. The needs assessment therefore represents the perspectives of practitioners directly involved in supporting numeracy learning in inclusive classrooms.

Three students were included in the structured classroom observations as illustrative cases representing three learner profiles: one regular student, one slow learner, and one student with special educational needs. Because only three students were observed, these observations were used to contextualize the questionnaire findings rather than to estimate the prevalence of learner differences in the wider population.

### 2.3 Instruments and data collection

Two data collection instruments were used in the present needs assessment: a teacher needs-analysis questionnaire and a structured classroom observation sheet.

The teacher questionnaire consisted of 13 statements organized into four dimensions: (1) factual conditions of numeracy learning in inclusive classrooms (Items 1–4), (2) current implementation of Problem-Based Learning (Items 5–7), (3) implementation of differentiated instruction (Items 8–10), and (4) perceived urgency and need for a new instructional model (Items 11–13). Each item was rated on a four-point Likert scale: 1 = strongly disagree, 2 = disagree, 3 = agree, and 4 = strongly agree. The four-point response format was used to avoid a neutral midpoint and require respondents to indicate the direction of their judgment.

Before data collection, the questionnaire was reviewed by three experts with relevant expertise in mathematics education and inclusive education. Content validity was assessed using Aiken’s V based on the experts’ ratings. The results showed that all 13 items obtained Aiken’s V values above the predetermined minimum criterion and were therefore considered valid for use in the needs assessment. This expert-validation process was conducted to examine the relevance and clarity of the questionnaire items before administration to the participating teachers.

Internal consistency was subsequently examined using Cronbach’s alpha based on responses from the 20 participating teachers. The overall 13-item questionnaire yielded a Cronbach’s alpha of 0.79. Dimension-level coefficients were 0.65 for factual conditions, 0.58 for PBL implementation, 0.18 for differentiated instruction, and 0.00 for the perceived urgency and need dimension. These dimension-level coefficients should be interpreted cautiously because of the small sample size (n = 20), the limited number of items within each dimension, and the absence of response variation for Items 12 and 13. Accordingly, the questionnaire was used primarily as a descriptive needs-assessment instrument, rather than as a psychometrically established multidimensional scale.

The structured classroom observation sheet assessed four areas: contextual problem solving, mathematical representation, instructional support required, and classroom participation. The observation instrument used a four-point rating scale ranging from very limited to independent. The observations of three students representing different learner profiles were used as illustrative cases to contextualize the quantitative questionnaire findings, rather than as a basis for statistical generalization.

### 2.4 Data analysis

Questionnaire responses were analyzed using descriptive statistics at the item level. For each item, the mean, standard deviation, and frequency of responses in each Likert category were calculated. Because the sample consisted of 20 teachers, response frequencies and percentages were retained to show the distribution of judgments.

The primary descriptive statistic was the raw Likert mean, ranging from 1 to 4. Higher mean scores indicated stronger endorsement of the statement. For interpretive purposes, responses in categories 3 and 4 were also summarized as positive responses because both categories represented agreement with the corresponding statement.

The 0–100 indicator scores reported in the previous version of the manuscript were not interpreted as percentages of teachers agreeing. Those values were transformed scores derived from the original scoring table and could be misleading because they do not represent the proportion of respondents selecting a particular response category. Therefore, the revised manuscript reports raw means, standard deviations, and actual response frequencies as the primary evidence.

Student observation data were summarized descriptively across the three illustrative learner profiles. The observations were used to provide contextual examples of differences in problem-solving independence, mathematical representation, instructional support, and classroom participation. No inferential analysis or population-level generalization was conducted from these three cases.

Finally, questionnaire findings and illustrative observations were synthesized into instructional design requirements. The synthesis was treated as an interpretive design step rather than as a formal root-cause analysis. Accordingly, the term “underlying instructional factors” is used instead of “critical root causes.

## 3. Results and discussion

The needs assessment results are presented first at the item level to provide an overall description of teachers’ responses before examining the four thematic dimensions of the questionnaire. The questionnaire used a four-point Likert scale, ranging from 1 (not needed) to 4 (strongly needed and urgent). Table 1 presents the mean, standard deviation (SD), frequency of responses for each response category, and the proportion of respondents selecting scores 3 or 4. This presentation allows the distribution of responses to be examined directly rather than relying on transformed percentage scores.

**Table 1.** Item-level descriptive statistics of the teacher needs-assessment questionnaire.

Item	Mean	SD	Score 1	Score 2	Score 3	Score 4	Score 3–4
1	3.60	0.50	0	0	8	12	100%
2	3.60	0.50	0	0	8	12	100%
3	3.70	0.47	0	0	6	14	100%
4	3.40	0.50	0	0	12	8	100%
5	3.30	0.57	0	1	12	7	95%
6	3.55	0.51	0	0	9	11	100%
7	3.70	0.47	0	0	6	14	100%
8	2.85	0.49	0	4	15	1	80%
9	3.70	0.47	0	0	6	14	100%
10	3.85	0.37	0	0	3	17	100%
11	3.95	0.22	0	0	1	19	100%
12	4.00	0.00	0	0	0	20	100%
13	4.00	0.00	0	0	0	20	100%

Note. Score 1 = Not Needed; Score 2 = Quite Needed; Score 3 = Needed; Score 4 = Strongly Needed and Urgent. Score 3–4 represents the proportion of teachers selecting the two higher response categories. N = 20.

Overall, the responses indicate a consistently high level of perceived need across most questionnaire items. The item means ranged from 2.85 to 4.00, while the proportion of respondents selecting scores 3 or 4 ranged from 80% to 100%. The highest mean scores were observed for Items 12 and 13 (M = 4.00, SD = 0.00), followed by Item 11 (M = 3.95, SD = 0.22). These items concerned the need for an operational teacher guide, the urgency of developing the DIF-PBL model, and the need for a numeracy learning model combining PBL with practical differentiation strategies. In contrast, Item 8 had the lowest mean (M = 2.85, SD = 0.49), although 80% of respondents still selected scores 3 or 4, indicating that difficulty in implementing differentiated learning was present for most respondents but was less uniformly endorsed than the other needs.

The results are further organized below into four dimensions: (1) current conditions of numeracy learning in inclusive classrooms, (2) implementation of problem-based learning, (3) differentiated instruction, and (4) perceived need for an integrated instructional model.

### 3.1 Current conditions of numeracy learning in inclusive classrooms

The first dimension examined teachers’ perceptions of current numeracy-learning conditions in inclusive classrooms. The four items produced a dimension mean of 3.58 (SD = 0.50). All 20 teachers selected either agree or strongly agree for each of the four statements.

The highest mean was observed for Item 3 (M = 3.70, SD = 0.47), concerning students with special educational needs appearing anxious, passive, or frustrated when faced with abstract numeracy problems. Fourteen teachers selected strongly agree and six selected agree. Items 1 and 2 both obtained a mean of 3.60 (SD = 0.50), with all respondents agreeing that disparities in basic numeracy skills were present and that balancing instructional time and attention across learners was difficult. Item 4 obtained the lowest mean within this dimension (M = 3.40, SD = 0.50), although all respondents still agreed or strongly agreed that current assessment instruments did not fully accommodate differences in students’ cognitive abilities.

These findings indicate that teachers perceived learner heterogeneity and the associated instructional challenges as important characteristics of numeracy learning in the participating inclusive classrooms.

### 3.2 Current implementation of Problem-Based Learning

The three items examining conventional Problem-Based Learning produced a dimension mean of 3.52 (SD = 0.54). Item 7 obtained the highest mean (M = 3.70, SD = 0.47), with all 20 teachers agreeing or strongly agreeing that the PBL steps required modification to make instructions easier to understand for students with different levels of learning readiness. Item 6 also received strong endorsement (M = 3.55, SD = 0.51), with all respondents agreeing or strongly agreeing that fast learners tended to dominate regular PBL group activities. Item 5 obtained a mean of 3.30 (SD = 0.57), with 19 of the 20 respondents agreeing or strongly agreeing that conventional PBL was difficult to implement effectively for slow learners.

These findings suggest that the main concern was not the rejection of PBL itself, but the perceived need to make its procedures more flexible and accessible to learners with different levels of readiness. The finding concerning unequal participation also indicates a need for more structured collaborative arrangements within inclusive PBL activities.

### 3.3 Challenges in Implementing Differentiated Instruction

The three items addressing differentiated instruction produced a dimension mean of 3.47 (SD = 0.62). The strongest response concerned the use of gradual learning representations, from concrete objects to pictures and numerical symbols (Item 10: M = 3.85, SD = 0.37). All 20 teachers agreed or strongly agreed with this statement, including 17 who selected strongly agree.

Item 9, concerning the administrative workload associated with preparing differentiated learning plans, also received strong endorsement (M = 3.70, SD = 0.47), with all respondents selecting agree or strongly agree. Item 8 differed from the other items because it described a difficulty, rather than confidence, in implementing differentiated instruction. The item obtained a mean of 2.85 (SD = 0.49), with 15 teachers agreeing and one strongly agreeing that they experienced technical difficulty implementing differentiated instruction, while four selected disagree. Thus, 80% of respondents endorsed the statement that they experienced implementation difficulties. The score should not be interpreted as 66.25% teacher confidence, as stated in the previous version of the manuscript.

Overall, the findings indicate that teachers recognized the need for differentiated instruction but also experienced practical difficulties in translating differentiation principles into classroom planning, materials, and instructional procedures.

### 3.4 Perceived need for an integrated DIF-PBL model

The final three items examined teachers’ perceived need for an integrated instructional model. This dimension showed the highest mean score among the four dimensions (M = 3.98, SD = 0.13).

Item 11, concerning the need for a numeracy learning model that combines Problem-Based Learning with practical differentiation strategies, obtained a mean of 3.95 (SD = 0.22). Nineteen teachers selected strongly agree and one selected agree. Item 12, concerning the need for an operational teacher guide containing practical examples, worksheets, and inclusive assessment rubrics, obtained a mean of 4.00 (SD = 0.00), with all 20 teachers selecting strongly agree. Item 13, which explicitly referred to the urgency of developing the DIF-PBL model, also obtained a mean of 4.00 (SD = 0.00), with all respondents selecting strongly agree.

These results indicate a very strong perceived need for practical instructional support and for an integrated approach combining PBL and differentiated instruction. However, because Items 11–13 directly frame the proposed model and its urgency, these responses should be interpreted as evidence of perceived need, rather than independent evidence that the DIF-PBL model itself is effective or superior to existing approaches.

### 3.5 Illustrative observations of learner differences

Structured observations were conducted with three students representing different learner profiles: one regular student, one slow learner, and one student with special educational needs. The observation instrument covered contextual problem solving, mathematical representation, instructional support required, and classroom participation.

The observations provided illustrative evidence of variation in learning readiness and instructional support needs. The regular student demonstrated greater independence in solving contextual numeracy tasks, whereas the slow learner required additional processing time and repeated guidance. The student with special educational needs relied more heavily on concrete materials, visual representations, and teacher scaffolding during the observed activities.

These observations are interpreted only as illustrative cases. Given the very small number of observed students, they cannot be used to estimate the prevalence of these characteristics or to establish general differences between regular students, slow learners, and students with special educational needs. Their primary function in this study was to provide contextual information that helped interpret the teacher questionnaire findings.

### 3.6 Synthesis of the needs assessment

**From empirical findings to preliminary instructional requirements**

As shown in Table 2, the needs assessment points to several recurring instructional requirements. First, learner heterogeneity requires differentiated learning pathways, tiered tasks, and flexible teacher support. Second, the reported difficulty with abstract numeracy and the observed differences in students’ learning needs indicate the importance of a gradual concrete–pictorial–abstract progression accompanied by systematic scaffolding. Third, teachers’ responses regarding conventional PBL suggest a need for flexible procedures, structured participation, and opportunities to modify the PBL process according to learner readiness. Finally, the responses concerning differentiated instruction, planning workload, teacher guidance, and the integration of PBL with differentiation highlight the need for practical implementation support. Collectively, these findings informed the preliminary design implications of DIF-PBL presented in the subsequent section.

**Table 2.** Traceability from empirical findings to preliminary instructional requirements and DIF-PBL design implications.

Empirical finding	Evidence source	Instructional requirement	Proposed DIF-PBL implication
Differences in numeracy readiness	Items 1–3; student observations	Differentiated learning pathways	Tiered learning tasks and differentiated pathways
Difficulty balancing instructional support	Item 2	Flexible instructional support	Adaptive teacher support within PBL
Difficulty with abstract numeracy	Item 3; observations	Gradual conceptual progression	CPA sequence and scaffolding
Assessment does not accommodate learner differences	Item 4	Adaptive assessment	Differentiated formative assessment
Conventional PBL is difficult for some learners	Item 5	Flexible PBL procedures	Modified PBL syntax
Unequal participation in PBL groups	Item 6	Structured collaborative participation	Explicit group roles and participation structures
PBL syntax requires modification	Item 7	Flexible PBL syntax	Adaptive decision points
Teachers experience difficulty implementing DI	Item 8	Operational guidance	Practical teacher guidance and examples
Differentiated planning increases workload	Item 9	Integrated planning support	Integrated planning templates
Need for concrete–pictorial–symbolic progression	Item 10	CPA progression	CPA-based learning sequence
Need for integrated PBL + differentiation	Item 11	Integrated instructional framework	DIF-PBL conceptual integration
Need for operational teacher guidance	Item 12	Implementation support	Teacher guide, worksheets, rubrics
Strongly stated urgency for DIF-PBL	Item 13	Perceived demand	Basis for prototype development

## 4. Discussion

### 4.1 Why inclusive numeracy learning requires adaptive instruction

The findings of this study indicate that numeracy learning in inclusive elementary classrooms is characterized by substantial heterogeneity in students’ learning readiness, mathematical understanding, and instructional support needs. Teachers consistently reported difficulties in simultaneously facilitating students with diverse cognitive abilities while ensuring equitable participation in classroom learning. These findings reinforce the fundamental principle of inclusive education that instructional practices should be designed to accommodate learner diversity rather than expecting students to adapt to uniform teaching approaches ( Dr. Ranbir, 2024; Kaur & Bhatia, 2024; Letzel-Alt & Pozas, 2025).

From a cognitive development perspective, Piaget’s theory suggests that elementary school students are predominantly situated within the concrete operational stage, during which mathematical understanding develops most effectively through direct manipulation of concrete objects before progressing toward symbolic abstraction ( Wadsworth, 1995). However, students with special educational needs frequently require extended experiences with concrete representations and additional instructional support before achieving conceptual understanding comparable to their peers ( Flores et al., 2023; Flores & Hinton, 2022). Consequently, identical instructional activities may produce substantially different learning outcomes among students within the same classroom.

The present findings are also consistent with Tomlinson’s theory of Differentiated Instruction, which emphasizes that effective teaching should be responsive to students’ readiness, interests, and learning profiles ( Economidou Stavrou, 2024; Tomlinson, 2021). Teachers participating in this study recognized the necessity of adapting learning activities according to students’ diverse characteristics, yet they simultaneously acknowledged experiencing considerable challenges in implementing differentiation consistently. This discrepancy suggests that awareness of differentiation alone is insufficient; teachers require structured instructional frameworks that operationalize differentiation within daily classroom practice.

Furthermore, the findings support the principles of Universal Design for Learning (UDL), which advocate providing multiple means of engagement, representation, and action or expression to ensure equitable learning opportunities for all students. The reported disparities in numeracy achievement and the limitations of existing assessment practices indicate that inclusive numeracy learning requires instructional environments capable of accommodating diverse pathways toward achieving common learning objectives. Therefore, adaptive instruction should not be viewed as an optional teaching strategy but rather as a fundamental requirement for achieving educational equity in inclusive classrooms.

### 4.2 Why conventional problem-based learning falls short in inclusive classrooms

Although Problem-Based Learning has consistently demonstrated positive effects on students’ critical thinking ( Nguyen et al., 2025), collaboration ( Wijayanto et al., 2025), and mathematical problem-solving abilities ( Dorimana et al., 2022), the present findings reveal several limitations when conventional PBL is implemented within inclusive classrooms. Teachers reported that existing PBL procedures are insufficiently flexible to accommodate learners with diverse cognitive readiness and frequently result in unequal participation during collaborative learning activities.

One possible explanation can be understood through Cognitive Load Theory. Authentic contextual problems, while educationally meaningful, often require learners to simultaneously process multiple sources of information, activate prior knowledge, coordinate collaborative interactions, and construct appropriate solution strategies ( Evains et al., 2024; Lovellette et al., 2024). For students with limited working memory capacity or delayed cognitive development, these instructional demands may generate excessive cognitive load, thereby reducing learning effectiveness. Consequently, the complexity of conventional PBL tasks may unintentionally disadvantage students with special educational needs unless appropriate instructional supports are incorporated ( D’Elia et al., 2025; Riera Hermida et al., 2025).

The collaborative nature of PBL also presents unique challenges within heterogeneous classrooms. Collaborative learning theories emphasize that group interaction promotes knowledge construction through social negotiation and shared responsibility ( Hmelo-Silver & Barrows, 2008; Zabolotna et al., 2025). However, the findings indicate that fast learners frequently dominate discussions and decision-making processes, while students requiring greater instructional support become passive participants. Such unequal participation limits opportunities for meaningful engagement and may reinforce existing achievement disparities rather than reducing them.

These findings do not suggest that Problem-Based Learning is inappropriate for inclusive education. Instead, they indicate that the instructional syntax of conventional PBL requires systematic adaptation to ensure that inquiry, collaboration, and problem-solving activities remain accessible for students with varying learning characteristics. Consequently, the effectiveness of PBL within inclusive classrooms depends not only on authentic problems but also on the flexibility of instructional design supporting diverse learners throughout the learning process.

### 4.3 Instructional design implications emerging from the needs assessment

The needs assessment suggests several instructional design implications for a future DIF-PBL model. These implications should be understood as preliminary design requirements rather than empirically validated components of an effective model.

First, differentiated learning pathways and tiered learning tasks are indicated by the substantial variation in teachers’ reports of students’ numeracy readiness and the difficulty of balancing instructional support within the same classroom. A future model should therefore allow students to work toward common learning objectives through appropriately differentiated levels of task complexity and support.

Second, the findings support a more flexible PBL syntax. Teachers reported that conventional PBL was difficult for some learners and that its instructional steps required modification. The implication is not that PBL should be abandoned, but that its procedures should include opportunities for adjusting task demands, explanations, pacing, and support according to learner readiness.

Third, the findings support the inclusion of a Concrete–Pictorial–Abstract progression and systematic scaffolding. Teachers strongly endorsed the need for gradual movement from concrete materials to pictorial and symbolic representations, while the observations provided illustrative examples of students requiring different levels of representational and instructional support.

Fourth, the findings indicate a need for structured collaborative participation. Because teachers reported that faster learners could dominate group activities, future model development should include explicit group roles and participation structures. Peer tutoring may be considered as one possible implementation strategy, but it was not directly measured by the questionnaire and therefore should be treated as a design option rather than an empirically established requirement.

Fifth, adaptive assessment and practical teacher guidance are supported by the findings. Teachers reported limitations in current assessment instruments and strong demand for an operational teacher guide containing practical examples, worksheets, and inclusive assessment rubrics. These findings suggest that differentiation should extend beyond learning activities to include assessment and implementation support.

### 4.4 From empirical needs to preliminary DIF-PBL model

The findings provide an empirical starting point for the subsequent design of the DIF-PBL model. The proposed framework should not be interpreted as having been validated by the present needs assessment. Instead, the findings identify classroom conditions and teacher-perceived requirements that can be translated into an initial instructional specification.

The central design principle is the integration of Problem-Based Learning with differentiated instructional pathways. PBL provides the overall problem-solving structure, while differentiation is expected to regulate task complexity, representational support, pacing, and instructional assistance according to learner readiness. CPA progression and systematic scaffolding can provide additional support for learners who experience difficulty with abstract mathematical representations. Structured collaborative participation can be incorporated to reduce unequal participation during group activities, while adaptive assessment can provide teachers with information for adjusting subsequent learning activities.

These design implications require further validation through expert judgment, prototype testing, classroom implementation, and effectiveness studies. The present study therefore provides a needs-based design specification rather than evidence of the effectiveness of the DIF-PBL model.

### 4.5 Research contributions

This study contributes by linking teacher-reported instructional problems in inclusive numeracy classrooms to preliminary design implications for an integrated PBL and differentiated-instruction model. The study also demonstrates the importance of examining practical implementation issues, rather than focusing only on the theoretical compatibility of PBL and Differentiated Instruction. A further contribution is the explicit identification of practical implementation needs, including differentiated learning pathways, flexible PBL procedures, gradual mathematical representation, scaffolding, adaptive assessment, and operational teacher guidance. These findings provide a transparent empirical basis for subsequent prototype development and validation.

### 4.6 Limitations and future research

Several limitations should be considered when interpreting the findings. First, the questionnaire involved only 20 teachers from three inclusive elementary schools in one district, limiting the transferability of the findings to other educational contexts. Second, the internal consistency estimates, particularly at the dimension level, should be interpreted cautiously because of the small sample and the small number of items per dimension.

Third, several questionnaire items were explicitly framed around the proposed DIF-PBL model. In particular, Items 11 and 13 directly asked teachers about their need for an integrated model and the urgency of DIF-PBL development. Item 10 also directly specified a concrete–pictorial–symbolic progression. These formulations may introduce acquiescence and framing bias and create partial circularity between the needs assessment and the proposed design. The findings should therefore be interpreted as evidence of perceived instructional need rather than independent confirmation of the proposed model components.

Fourth, the researchers were involved in developing the DIF-PBL model, which creates a potential dual-role bias in interpreting teachers’ perceived needs. Future studies should strengthen independence through externally reviewed instruments, blinded analysis where feasible, and validation by researchers who are not directly involved in model development.

Fifth, the student observations involved only three illustrative cases. The available observation records provide contextual information about learner profiles and instructional support needs, but the small number of cases does not permit generalization. Future studies should include larger and more systematically sampled groups of students and report grade level, learner identification procedures, observation duration, number of sessions, observer characteristics, and task specifications in greater detail.

Finally, the present study does not evaluate the effectiveness of DIF-PBL. Subsequent research should examine the validity, practicality, implementation fidelity, and effectiveness of the resulting prototype across multiple inclusive elementary-school contexts.

## 5. Conclusion

This study examined teachers’ perceived instructional needs as the empirical starting point for designing a Differentiation-Based Problem-Based Learning (DIF-PBL) model for inclusive numeracy learning. The questionnaire findings indicate substantial perceived challenges related to learner heterogeneity, balancing instructional support, conventional PBL implementation, differentiated instruction, and assessment practices.

The strongest responses concerned the need for an integrated PBL and differentiation approach, an operational teacher guide, and the perceived urgency of developing the proposed DIF-PBL model. The findings also indicate practical requirements for differentiated learning pathways, flexible PBL procedures, gradual concrete–pictorial–symbolic representation, systematic scaffolding, structured collaborative participation, and adaptive assessment.

The three student observations provided illustrative contextual evidence of variation in learning readiness, mathematical representation, instructional support, and classroom participation. Because these observations involved only three students, they should not be interpreted as evidence of population-level differences.

Overall, the study provides a transparent needs-based foundation for subsequent DIF-PBL prototype development. However, the findings do not establish the effectiveness or superiority of the proposed model. Expert validation, prototype testing, classroom implementation, and effectiveness evaluation are required in subsequent research.

## Ethical approval

This study was conducted in accordance with the principles of the Declaration of Helsinki. Ethical approval was obtained from the Institutional Review Board (IRB) of Universitas Bosowa (Approval No. 203/1.02/DRIPM-UNIBOS/VII/2025; approved on 21 July 2025).

## AI statement

The authors stated that generative artificial intelligence tools were used in a limited manner to assist with language editing, organization of academic writing, improvement of clarity, and improving image quality. The conceptual framework, research design, data analysis, interpretation, and final conclusions were developed entirely by the authors.

## Informed consent

Written informed consent was obtained from all participating teachers. For student participants who were minors, written informed consent was obtained from their parents or legal guardians prior to participation. Assent was also obtained from the students according to their level of understanding.

## Data Availability

The data supporting the findings of this study are available in figshare:
• Teacher Needs Analysis Instrument. DOI: https://doi.org/10.6084/m9.figshare.33092594.v1 ( Putri, 2026a) This repository contains the teacher needs analysis questionnaire, anonymized raw responses from 20 participating teachers, and the descriptive data used to generate the quantitative findings reported in this study.
• Structured Classroom Observation Sheet. DOI: https://doi.org/10.6084/m9.figshare.33130376 ( Putri, 2026b). This repository contains the structured classroom observation instrument together with anonymized observation summaries for the three learner profiles (regular student, slow learner, and student with special educational needs) used during the initial needs assessment. All datasets have been anonymized to protect participant confidentiality. Data are available under the terms of the Creative Commons Attribution 4.0 International (CC-BY 4.0) license.
