# Supplementary material for: From Needs Assessment to Instructional Design: Empirical Requirements for a DIF-PBL Model in Inclusive Numeracy Learning
Source: F1000Res. 2026 Nov 4;15:1482. Originally published 2026 Sep 3. [Version 2] doi: 10.12688/f1000research.187953.2 (PMC13740461; doi:10.12688/f1000research.187953.2)
Supplement: Peer review history

# Reviewer report: Reviewer response for version 2

**DOI:** 10.5256/f1000research.211866.r537972

**Published:** 2026-11-06

**Authors:** Andi Hasdiansyah

**Affiliations:** 1 Master of Pedagogy Program, Graduate School, Universitas Muhammadiyah Parepare, Parepare, Indonesia

I appreciate the authors' efforts in revising the manuscript and responding to the comments raised during the previous rounds of peer review.

 The second revised version satisfactorily addresses my principal concerns. The revisions have improved the clarity, coherence, and overall presentation of the manuscript. I am satisfied with the authors' responses and consider the manuscript to be in an acceptable form.

 I have no further substantive comments or requests for revision. I therefore recommend **Approved** for the current version of the manuscript.

 I thank the authors for their constructive engagement throughout the review process.

Is the work clearly and accurately presented and does it cite the current literature?

Partly

If applicable, is the statistical analysis and its interpretation appropriate?

Partly

Are all the source data underlying the results available to ensure full reproducibility?

Yes

Is the study design appropriate and is the work technically sound?

Yes

Are the conclusions drawn adequately supported by the results?

Yes

Are sufficient details of methods and analysis provided to allow replication by others?

Partly

Reviewer Expertise:

Education

I confirm that I have read this submission and believe that I have an appropriate level of expertise to confirm that it is of an acceptable scientific standard.

# Reviewer report: Reviewer response for version 2

**DOI:** 10.5256/f1000research.211866.r537971

**Published:** 2026-11-05

**Authors:** Marisol Jane M Beray

**Affiliations:** 1 Department of Applied Technology Education, Caraga State University (Ringgold ID: 489365), Cabadbaran City, Caraga, Philippines

I have no further comments.

Is the work clearly and accurately presented and does it cite the current literature?

Yes

If applicable, is the statistical analysis and its interpretation appropriate?

Partly

Are all the source data underlying the results available to ensure full reproducibility?

Partly

Is the study design appropriate and is the work technically sound?

Yes

Are the conclusions drawn adequately supported by the results?

Partly

Are sufficient details of methods and analysis provided to allow replication by others?

Partly

Reviewer Expertise:

Technology Education, Electronics Engineering Technology, Teacher Education

I confirm that I have read this submission and believe that I have an appropriate level of expertise to confirm that it is of an acceptable scientific standard.

# Reviewer report: Reviewer response for version 1

**DOI:** 10.5256/f1000research.207432.r518284

**Published:** 2026-09-28

**Authors:** Marisol Jane M Beray

**Affiliations:** 1 Department of Applied Technology Education, Caraga State University (Ringgold ID: 489365), Cabadbaran City, Caraga, Philippines

**Summary:**

 The authors describe the first phase of a larger R&D project aimed at building a Differentiation-Based Problem-Based Learning (DIF-PBL) model for numeracy in inclusive elementary classrooms. Twenty teachers filled out a 13-item, four-point Likert questionnaire, and three students (a regular student, a slow learner and a student with special educational needs) were observed. The findings are pulled together in a synthesis matrix, from which the authors derive design requirements for the model: CPA sequences, scaffolding, tiered tasks, peer tutoring, flexible PBL syntax, and adaptive assessment.

**Good Points:**

 This is a worthwhile topic. PBL and differentiated instruction are usually studied apart from each other, and starting model development from a needs assessment rather than from theory alone is a sensible move. The paper is easy to follow, and the line from research questions to results to design requirements is clear. I also appreciated that the authors deposited the questionnaire, anonymized responses and observation instruments in an open repository, and that the limitations section is honest about the single region, the teacher-heavy perspective and the early stage of the project.

**Main concerns**

**1. **I could not make sense of the percentages. With only 20 respondents, figures like 87.5%, 83.75% and 91.25% can't be proportions of teachers (87.5% of 20 would be 17.5 people). They look like normalized Likert scores, yet the figures are titled "Percentage of Teachers Agreeing" and the text says things like "87.50% of teachers agreed." Please spell out the formula, retitle the figures, reword the text, and give item means, SDs and the actual agree/disagree counts.

**2**. Figure 4 and Section 3.3 say opposite things. The figure describes the 66.25% item as "difficulty in implementing differentiated instruction," but the text reports that "only 66.25% indicated confidence." Those are very different readings. Since this item feeds into the 81.67% dimension mean and supports the argument that teachers need practical guidance, I'd ask the authors to go back to the original item wording and scoring direction and fix whichever is wrong.

**3**. The "mixed-methods" label seems too strong. I couldn't find any qualitative teacher data (interviews, focus groups, open-ended items). The only qualitative strand is the observation of three students, and Section 2.3 promises "three techniques" but describes two. Calling this a convergent parallel design with methodological triangulation isn't really justified. The authors could either report qualitative teacher data or describe the study as a descriptive survey with illustrative case observations.

 4. The "critical root causes" don't have a clear analytical basis. In Table 1, that column mostly restates the findings, and no root-cause procedure (five-whys, fishbone, coded qualitative data, etc.) is described. I'd suggest either explaining the method or using more modest wording such as "underlying instructional factors." Root causes are also named in the aims but have no matching research question.

 5. I couldn't trace the design requirements back to the data. The synthesis lists four requirements, while the abstract and conclusion list seven components. Peer tutoring and adaptive assessment in particular seem thinly supported, since no questionnaire item asks about peer tutoring directly. A simple traceability table (finding → requirement → component) would clear this up.

 6. A few questionnaire items are leading or circular. "Development of the DIF-PBL model is urgently needed" names the authors' own model, and the item on gradual media from concrete to symbolic is essentially CPA, which is later presented as an empirical finding. The 93–95% urgency scores are therefore open to acquiescence bias, which the paper doesn't mention. Because the authors are also the model's developers, that dual role deserves a line in the limitations too.

 7. There isn't enough methodological detail to replicate the study. It would help to see:

- the actual Cronbach's alpha (ideally per dimension, with a note that n = 20 makes it unstable);
- quantitative expert-validation results (CVI or Aiken's V);
- the full wording of all 13 items;
- the number of schools and how they were selected;
- teacher characteristics (grade taught, experience, inclusive-education training);
- how the teachers were recruited.

8. The student observations are under-described. Please tell us the grade level, the type of SEN and how it was identified, how "slow learner" was defined, what tasks were used, how many sessions there were and how long they lasted, who observed, and whether a rubric was used. With n = 3, these read best as illustrative cases; I'd avoid framing them as evidence of disparities.

**Small points**

- Some citations don't seem to back up the claims they're attached to. For example, Yeh et al. (2019) is cited for Indonesian national data, and Evains et al. (2024), a physics and smartphone study, is cited for SEN and cognitive load. Foundational sources such as Tomlinson, Sweller, Vygotsky and CAST/UDL would be better cited directly, and the AKM data should be updated.
- Section 3 is titled "Results and discussion," but Section 4 is already the Discussion.
- Words like "comprehensive," "evidence-based" and "replicable framework" go further than 20 questionnaires and three observations can carry. I'd tone them down.
- A few typos and formatting slips: "The The" in a reference, the "Dr. Ranbir" citation format, a double comma in affiliation 1, and incomplete CRediT roles.
- Please share the raw observation records and a codebook, not just summaries.

**Overall**

 This is a useful exploratory foundation, and I don't think any of the above requires new data collection (unless the authors want to add teacher qualitative data). That said, the percentage problem, the Figure 4 contradiction, the overstated design claims, and the missing methodological detail currently keep the conclusions from being fully supported.

 I'd want points 1–5 and 7–8 addressed before the conclusions are considered sound.

Is the work clearly and accurately presented and does it cite the current literature?

Yes

If applicable, is the statistical analysis and its interpretation appropriate?

Partly

Are all the source data underlying the results available to ensure full reproducibility?

Partly

Is the study design appropriate and is the work technically sound?

Yes

Are the conclusions drawn adequately supported by the results?

Partly

Are sufficient details of methods and analysis provided to allow replication by others?

Partly

Reviewer Expertise:

Technology Education, Electronics Engineering Technology, Teacher Education

I confirm that I have read this submission and believe that I have an appropriate level of expertise to confirm that it is of an acceptable scientific standard, however I have significant reservations, as outlined above.

# Response

**Published:** 2026-10-01

**Authors:** Mentari Rama Putri

**Affiliations:** Postgraduate Program, State University of Makassar, Makassar, South Sulawesi, Indonesia

We sincerely thank the reviewer for the constructive and detailed evaluation of our manuscript. We appreciate the positive assessment of the relevance of the topic, the integration of Problem-Based Learning (PBL) and differentiated instruction, the needs-assessment approach, and the open availability of the research materials. We have carefully considered all comments and substantially revised the manuscript. The major revisions concern the presentation and interpretation of the questionnaire data, clarification of the research design, refinement of the instructional design claims, additional methodological information, and clearer reporting of the illustrative student observations.

 Our responses to each comment are provided below.

 Comment 1. Percentage scores are difficult to interpret

 I could not make sense of the percentages. With only 20 respondents, figures like 87.5%, 83.75% and 91.25% can't be proportions of teachers... Please spell out the formula, retitle the figures, reword the text, and give item means, SDs and the actual agree/disagree counts.

 Response

 Thank you for identifying this important issue. We agree that the previous presentation could incorrectly be interpreted as the percentage of teachers agreeing with each statement. The transformed values previously reported in Figures 2–5 were derived from Likert-score calculations and did not represent the proportion of respondents.

 To eliminate this ambiguity, we have substantially revised the presentation of the questionnaire results. Figures 2–5 have been removed and replaced with a new item-level descriptive statistics table (Table 1) reporting the mean, standard deviation, response frequencies for Scores 1–4, and the proportion of respondents selecting Scores 3–4.

 The Results section has also been rewritten so that statements such as “87.50% of teachers agreed” are no longer used. Instead, the findings are reported using the actual response distribution and descriptive statistics. For example, Item 8 is now reported as *M* = 2.85, *SD* = 0.49, with 15 respondents selecting Score 3 and one selecting Score 4, corresponding to 80% selecting the two higher response categories.

 This revision makes the numerical interpretation transparent and directly verifiable from the response frequencies.

 Changes in manuscript: Results section, new Table 1, and corresponding interpretation of Sections 3.1–3.4.

 Comment 2. Contradiction concerning Item 8

 Figure 4 and Section 3.3 say opposite things... The figure describes the 66.25% item as “difficulty in implementing differentiated instruction,” but the text reports that “only 66.25% indicated confidence.”

 Response

 We agree with the reviewer. The original wording incorrectly interpreted Item 8 as measuring teacher confidence. The original questionnaire item actually measures teachers’ perceived difficulty in implementing differentiated instruction technically.

 We have therefore corrected the interpretation throughout the manuscript. In the revised Results section, Item 8 is reported as *M* = 2.85, *SD* = 0.49. Fifteen respondents selected Score 3 and one selected Score 4, meaning that 80% of respondents endorsed the statement at the two higher response categories.

 The revised text explicitly states that this finding represents a perceived implementation challenge, rather than evidence of teacher confidence.

 The previous figure containing the ambiguous transformed percentage has also been removed.

 Changes in manuscript: Section 3.3 and Table 1.

 Comment 3. The mixed-methods label is too strong

 The “mixed-methods” label seems too strong... The authors could either report qualitative teacher data or describe the study as a descriptive survey with illustrative case observations.

 Response

 We agree with this comment. Upon re-examination, the present article does not contain a qualitative teacher strand such as interviews, focus groups, or open-ended questionnaire responses. The primary dataset is the quantitative teacher needs-assessment questionnaire, complemented by observations of three students.

 Accordingly, we have removed the “mixed-methods convergent parallel design” terminology and no longer describe the study as a convergent parallel mixed-methods study or as involving methodological triangulation.

 The revised study is described as a descriptive needs assessment within the initial phase of a broader Research and Development project, complemented by illustrative student observations. The role of the student observations is explicitly limited to providing contextual illustrations rather than constituting a separate qualitative strand for methodological integration.

 We have also revised the description of the data collection procedures so that the number and type of data-collection approaches are consistent throughout the manuscript.

 Changes in manuscript: Abstract, Introduction, Sections 2.1–2.3, Results, and Discussion.

 Comment 4. The “critical root causes” lack a clear analytical basis

 The “critical root causes” don't have a clear analytical basis... I'd suggest either explaining the method or using more modest wording such as “underlying instructional factors.”

 Response

 We agree. The previous use of the term “critical root causes” implied a formal root-cause analysis that was not actually conducted using a structured procedure such as five-whys, fishbone analysis, or qualitative coding.

 We have therefore replaced this terminology with “underlying instructional factors” where appropriate. We have also removed “critical root causes” from the stated aims and revised the research questions accordingly.

 The revised analysis focuses on identifying empirical findings, the evidence supporting those findings, the corresponding instructional requirements, and their preliminary implications for DIF-PBL design. This better reflects the actual analytical procedure used in the study.

 Changes in manuscript: Abstract, Introduction, Results synthesis, Table 2, Discussion, and Conclusion.

 Comment 5. The design requirements cannot be clearly traced back to the data

 The synthesis lists four requirements, while the abstract and conclusion list seven components. Peer tutoring and adaptive assessment in particular seem thinly supported...

 Response

 Thank you. We agree that the previous manuscript did not provide sufficient traceability between individual empirical findings and the proposed design implications.

 To address this issue, we have added a new Table 2, “Traceability from empirical findings to preliminary instructional requirements and DIF-PBL design implications.” The table explicitly connects:

 Empirical finding → Evidence source → Instructional requirement → Proposed DIF-PBL implication.

 For example, the reported difficulty with abstract numeracy (Item 3 and the illustrative observations) is linked to the requirement for gradual conceptual progression and the preliminary implication of a Concrete–Pictorial–Abstract (CPA) sequence and scaffolding. Similarly, Item 4 directly informs the preliminary requirement for adaptive assessment.

 We have also revised our terminology. Rather than presenting all resulting elements as “empirically established components” of DIF-PBL, we now refer to them as preliminary instructional requirements or preliminary design implications derived from the needs assessment.

 We agree specifically regarding peer tutoring. Because no questionnaire item directly assessed the need for peer tutoring, it is no longer presented as a directly established empirical requirement. Where peer tutoring is discussed, it is framed as a possible implementation strategy rather than a direct finding of the needs assessment.

 Changes in manuscript: New Table 2 and corresponding revisions to the Abstract, Results, Discussion, and Conclusion.

 Comment 6. Some questionnaire items are leading or circular

 “Development of the DIF-PBL model is urgently needed” names the authors' own model... The 93–95% urgency scores are therefore open to acquiescence bias... the authors are also the model's developers.

 Response

 We appreciate this important methodological observation and agree that several items require cautious interpretation.

 In the revised manuscript, we explicitly acknowledge the potential for acquiescence and framing bias, particularly for Items 11–13, which directly refer to the need for an integrated PBL–differentiation model, an operational guidebook, and the urgency of developing DIF-PBL. We also acknowledge that Item 10 embeds the concrete–pictorial–symbolic progression within the questionnaire statement and therefore should not be interpreted as an independent empirical discovery of CPA.

 The corresponding Results and Discussion sections have been revised so that these items are interpreted as teachers’ perceived needs, rather than independent evidence establishing the superiority or effectiveness of the proposed model.

 We have also added the researchers’ dual role as developers of the DIF-PBL model as a limitation. This is now discussed alongside the small sample, the limited number of schools, and the exploratory nature of the needs assessment.

 Changes in manuscript: Methods, Results, Limitations, and Conclusion.

 Comment 7. Insufficient methodological detail

 It would help to see: the actual Cronbach's alpha... quantitative expert-validation results... full wording... number of schools... teacher characteristics... recruitment.

 Response

 We thank the reviewer for this suggestion. The Methods section has been expanded to improve methodological transparency and reproducibility.

 First, the revised manuscript now reports the internal-consistency analysis. The overall 13-item questionnaire yielded Cronbach’s α ≈ 0.79. Dimension-level coefficients were approximately 0.65 for Dimension 1, 0.58 for Dimension 2, 0.18 for Dimension 3, and 0.00 for Dimension 4. Because the questionnaire was administered to only 20 respondents and each dimension contains only a small number of items, we now explicitly describe these coefficients as exploratory and unstable estimates, rather than as evidence of an established psychometric scale.

 Second, we have clarified the content-validation procedure. The 13-item questionnaire was reviewed by three experts with relevant expertise in mathematics education and inclusive education, and content validity was assessed using Aiken’s V. All 13 items exceeded the predetermined minimum criterion and were therefore retained as valid for the needs assessment.

 Third, the complete questionnaire is already openly available in the research repository associated with the study, together with the anonymized responses from the 20 teachers. We have added an explicit statement directing readers to the repository rather than duplicating the complete instrument in an appendix.

 Fourth, the study setting has been clarified as three inclusive elementary schools in Tellu Siattinge District, Bone Regency, South Sulawesi, Indonesia. The three schools were selected purposively because they were inclusive schools serving learners with diverse educational needs and were the designated sites for the broader R&D project.

 Finally, we have clarified that the questionnaire respondents consisted of 20 classroom teachers and Special Education Support Teachers (GPK) involved in inclusive elementary education. We have avoided introducing teacher characteristics such as years of experience or grade taught where these were not systematically recorded in the dataset used for this article.

 Changes in manuscript: Sections 2.2–2.3, Data Availability Statement, and Limitations.

 Comment 8. Student observations are under-described

 Please tell us the grade level, type of SEN and how it was identified, how “slow learner” was defined, what tasks were used, how many sessions there were and how long they lasted, who observed, and whether a rubric was used. With n = 3, these read best as illustrative cases...

 Response

 We agree that the observations should be presented more cautiously and transparently.

 The revised manuscript now explicitly describes these observations as illustrative student cases, not as evidence from which population-level disparities can be generalized. The observations involved three learner profiles—a regular student, a slow learner, and a student with special educational needs—and focused on four areas: contextual problem solving, mathematical representation, instructional support required, and classroom participation. The structured observation instrument used a four-point rating scale ranging from very limited to independent and included observer notes.

 The observation materials are openly available in the research repository together with anonymized observation summaries for the three learner profiles. The observation task consisted of contextual numeracy problems, with attention to students’ ability to identify relevant information, select solution strategies, complete problem-solving steps, use concrete/pictorial/symbolic representations, and participate in classroom activities.

 We have also revised the manuscript to avoid presenting these three observations as evidence of general disparities. Instead, they are used to illustrate the types of learner differences and instructional support needs that were relevant to the needs-assessment phase.

 Where information such as formal diagnostic identification, exact observation duration, or detailed individual student characteristics was not systematically recorded in the needs-assessment dataset, we have avoided reconstructing or inferring such information retrospectively.

 Changes in manuscript: Section 2.3, Section 3.5, Discussion, Limitations, and Data Availability Statement.

 Small points

 Comment 9. Some citations do not support the claims

 Yeh et al. (2019)... Evains et al. (2024)... Foundational sources such as Tomlinson, Sweller, Vygotsky and CAST/UDL would be better cited directly, and the AKM data should be updated.

 Response

 We agree and have conducted a citation audit of the Introduction and Discussion. Citations that did not directly support the associated claims have been removed or replaced where appropriate. The revised manuscript also gives greater emphasis to foundational literature for differentiated instruction and instructional adaptation, rather than relying solely on secondary sources.

 Claims concerning national numeracy performance have also been revised so that they are supported by appropriate and up-to-date sources. We have avoided using sources from unrelated disciplinary contexts to support claims about inclusive numeracy learning.

 Changes in manuscript: Introduction and Discussion.

 Comment 10. Section 3 is titled “Results and discussion,” while Section 4 is already Discussion

 Response

 Agreed. Section 3 has been retitled “Results” to provide a clear separation between the empirical findings and the subsequent interpretation in Section 4, “Discussion.”

 Changes in manuscript: Section headings.

 Comment 11. Terms such as “comprehensive,” “evidence-based,” and “replicable framework” overstate the evidence

 Response

 We agree and have moderated these claims throughout the manuscript. In particular, terms such as “comprehensive,” “evidence-based,” and “replicable framework” have been replaced or qualified where they could imply a stronger evidentiary basis than is supported by 20 teacher responses and three illustrative student observations.

 The revised manuscript now describes the study as an exploratory needs assessment and refers to the resulting instructional implications as preliminary design implications rather than validated model components.

 Changes in manuscript: Abstract, Introduction, Results, Discussion, and Conclusion.

 Comment 12. Typos, formatting, and CRediT roles

 Response

 Thank you. We have carefully proofread the revised manuscript and corrected the identified typographical and formatting issues, including the duplicated “The The,” the formatting of the “Dr. Ranbir” citation, the double comma in the first affiliation, and inconsistencies in the CRediT author-contribution statements.

 Changes in manuscript: References, author information, and CRediT statement.

 Comment 13. Please share raw observation records and a codebook

 Response

 We agree with the importance of transparency for the observation data. The revised Data Availability Statement identifies the repository containing the structured classroom observation instrument and anonymized observation summaries for the three learner profiles. The observation instrument specifies the rating scale, indicators, observer notes, and domains used in the observations.

 Because the observations involve only three identifiable learner profiles in specific inclusive school contexts, we have taken care to remove direct participant identifiers. We have also clarified in the manuscript that these observations are illustrative rather than generalizable evidence.

 Important: if the reviewer specifically requires the raw observation records and a separate codebook, we recommend uploading those materials to the existing observation repository before final submission, provided they can be adequately anonymized. We should not claim that raw records have been shared if the repository currently contains only the instrument and observation summaries.

 Overall response

 We appreciate the reviewer’s overall assessment that the study provides a useful exploratory foundation and that the principal issues can be addressed without collecting new data. The revised manuscript substantially narrows the claims to match the evidence available in this initial needs-assessment phase. In particular, the revised version no longer treats the study as a convergent parallel mixed-methods study, no longer interprets transformed Likert scores as percentages of teachers, and no longer presents all proposed DIF-PBL elements as empirically established components.

 The revised manuscript instead positions the study as a descriptive needs assessment that translates teacher-reported instructional needs and illustrative classroom observations into preliminary instructional requirements for the subsequent development of DIF-PBL. We believe these changes improve the transparency, methodological alignment, and evidentiary basis of the manuscript.

# Reviewer report: Reviewer response for version 1

**DOI:** 10.5256/f1000research.207432.r518282

**Published:** 2026-09-24

**Authors:** Harlinda Syofyan

**Affiliations:** 1 Universitas Esa Unggul, Jakarta, Indonesia

A needs assessment forming the initial phase of developing the DIF-PBL model: 20 inclusive elementary teachers completed a 13-item, four-point Likert questionnaire, and three students with different learner profiles were observed. The topic is relevant, the manuscript is well organised, and the data are shared openly.

 However, there are issues in the reporting of methods, the calculation of percentages and the interpretation of results. 

 ​​​​​​Must Be Addressed

**1. Figure 4 contradicts the text. **Figure 4 labels the item “difficulty in implementing differentiated instruction” (66.25%), while Section 3.3 reads it as “only 66.25% indicated confidence”. Check the original item wording in the dataset and correct one of them; the dimension mean of 81.67% is affected too. *[Figure 4, p. 8; Section 3.3, p. 9]*

**2. The percentage metric is mislabelled. **Values such as 87.50% are impossible as proportions of 20 teachers (= 17.5 persons) and appear to be normalised Likert scores. State the formula, retitle Figures 2–5, rephrase the text accordingly, and report item means, SDs and actual frequencies. *[Figures 2–5, pp. 7–9; Sections 3.1–3.4; Section 2.4, p. 6]*

**3. The mixed-methods claim is overstated. **No qualitative teacher data (interviews, FGD, open items) are reported; the only qualitative strand is the observation of three students, and Section 2.3 announces three techniques while describing two. Either report the teacher qualitative data or relabel the design as a descriptive survey with illustrative case observations. *[Sections 2.1 and 2.3, pp. 5–6; Section 3.5, p. 10; Abstract]*

**4. Psychometric evidence is incomplete. **Report the actual Cronbach’s alpha (per dimension, noting that n = 20 makes it unstable), quantitative expert-validation results (CVI or Aiken’s V), and the full wording of all 13 items as an appendix. *[Section 2.3, p. 6]*

**5. Questionnaire items are leading. **The item “development of DIF-PBL is urgently needed” names the authors’ own model, and the item on “gradual concrete–pictorial–symbolic media” is essentially CPA, which is later presented as an empirical finding. Acknowledge the acquiescence bias and this circularity in the Limitations. *[Figures 4–5, pp. 8–9; Section 4.3, p. 14]*

**6. Root causes and traceability. **The “Critical Root Cause” column restates the findings with no analytical procedure; describe the procedure or rename the column, add a traceability table (finding → requirement → component), and reconcile the four requirements in Section 3.6 with the seven components in the Abstract (peer tutoring and adaptive assessment are not supported by the data). *[Table 1, pp. 12–13; Section 3.6, p. 10; Abstract; Figure 6, p. 11]*

**7. Student observations are under-reported. **Specify grade level, the type of SEN and how it was identified, the definition of “slow learner”, the numeracy tasks, the number and duration of sessions, the observers and any rubric; with n = 3, present these as illustrative cases rather than evidence of disparities. *[Sections 2.2–2.3, p. 6; Section 3.5, p. 10]*

**8. Sampling lacks detail. **Report the number of schools and the selection criteria, teacher characteristics (grade taught, experience, training in inclusive education or DI, number and type of SEN students) and how the teachers were recruited. *[Section 2.2, p. 6]*

 Suggested

**9. Check the citations. **Several references do not support their claims (Yeh et al. 2019 for Indonesian data; Evains et al. 2024 for SEN and cognitive load; Moriña 2017 for primary school; Zeng et al. 2024 for group dominance; Wadsworth 1995 listed as the wrong source type). Add foundational sources (Tomlinson, Sweller, Bruner, Vygotsky, CAST/UDL), update the AKM data, and review existing Indonesian studies combining PBL and differentiated instruction. *[Introduction, pp. 3–4; Sections 4.1–4.3, pp. 11–15; References, pp. 17–19]*

**10. Structure. **Rename Section 3 “Results”, since Section 4 is already the Discussion, and add a research question covering the “critical root causes” named in the aims. *[Section 3 heading, p. 7; research questions, p. 5]*

**11. Tone down the conclusions. **“Comprehensive”, “evidence-based” and “replicable framework” go beyond what 20 perception questionnaires and three observations support; add self-report bias and the researchers’ dual role as model developers to the Limitations. *[Sections 4.5–4.6, p. 15; Conclusion, pp. 15–16]*

**12. Data and minor points. **Share the raw observation records and a codebook, not only summaries; fix “The The”, the “Dr. Ranbir” citation format, the double comma in affiliation 1, and complete the CRediT roles. *[Data availability, p. 16; References, pp. 17–18; affiliation 1, p. 1; author roles, p. 2]*

 A useful exploratory foundation. Points 1–8 need to be resolved before the conclusions can be considered adequately supported, and none of them requires new data collection unless the authors choose to report teacher qualitative data (point 3). No competing interests were disclosed.

Is the work clearly and accurately presented and does it cite the current literature?

Partly

If applicable, is the statistical analysis and its interpretation appropriate?

Partly

Are all the source data underlying the results available to ensure full reproducibility?

Partly

Is the study design appropriate and is the work technically sound?

Partly

Are the conclusions drawn adequately supported by the results?

Partly

Are sufficient details of methods and analysis provided to allow replication by others?

Partly

Reviewer Expertise:

Education

I confirm that I have read this submission and believe that I have an appropriate level of expertise to confirm that it is of an acceptable scientific standard, however I have significant reservations, as outlined above.

# Response

**Published:** 2026-10-01

**Authors:** Mentari Rama Putri

**Affiliations:** Postgraduate Program, State University of Makassar, Makassar, South Sulawesi, Indonesia

We sincerely thank the reviewer for the careful and detailed evaluation of our manuscript. We appreciate the recognition of the relevance of the topic, the organization of the manuscript, and the transparency of the shared research materials. We have carefully addressed all of the comments and substantially revised the manuscript to improve methodological clarity, statistical reporting, traceability between empirical findings and design implications, and the appropriate scope of the conclusions.

 We respond to each point below.

**Comment 1. Figure 4 contradicts the text**

 Figure 4 labels the item “difficulty in implementing differentiated instruction” (66.25%), while Section 3.3 reads it as “only 66.25% indicated confidence”. Check the original item wording in the dataset and correct one of them; the dimension mean of 81.67% is affected too.

**Response**

 We agree with the reviewer. The original interpretation of Item 8 was incorrect.

 The original questionnaire item concerns teachers’ difficulty in implementing differentiated instruction technically, rather than teachers’ confidence in implementing differentiated instruction. We have therefore corrected the interpretation throughout the manuscript.

 In the revised analysis, Item 8 has a mean score of 3.85? [DO NOT use this—see note below], and the response distribution is reported directly rather than through the previous transformed percentage. Specifically, 15 respondents selected Score 3 and one respondent selected Score 4, resulting in 80% of respondents selecting Scores 3–4. The four respondents selecting Score 2 are also reported explicitly.

 The previous figure has been removed, and the Results section now interprets Item 8 as evidence of a perceived implementation difficulty, rather than teacher confidence.

 Changes made: Section 3.3 and new Table 1.

 Important correction: For Item 8 the correct mean is M = 2.85, SD = 0.49, not 3.85. 

**Comment 2. The percentage metric is mislabelled**

 Values such as 87.50% are impossible as proportions of 20 teachers... State the formula, retitle Figures 2–5, rephrase the text accordingly, and report item means, SDs and actual frequencies.

**Response**

 Thank you for highlighting this issue. We agree that the previous presentation could lead readers to interpret the transformed values as percentages of teachers, which was not appropriate.

 Rather than retaining the previous transformed percentage presentation, we have removed Figures 2–5 and replaced them with a new item-level descriptive statistics table (Table 1). The revised table reports:

1. item mean;
2. standard deviation;
3. frequency of Scores 1–4; and
4. the proportion of respondents selecting Scores 3–4.

This presentation allows the reader to directly examine the distribution of the 20 teachers’ responses.

 The Results section has also been rewritten so that transformed values such as 87.50%, 91.25%, and 93.75% are no longer described as the “percentage of teachers agreeing.” The revised manuscript distinguishes clearly between Likert-score descriptive statistics and actual response proportions.

 Changes made: Section 2.4, new Table 1, and Sections 3.1–3.4. Figures 2–5 have been removed.

**Comment 3. The mixed-methods claim is overstated**

 No qualitative teacher data are reported... Either report the teacher qualitative data or relabel the design as a descriptive survey with illustrative case observations.

**Response**

 We agree with the reviewer. The present article does not report interviews, focus groups, or open-ended teacher responses. The primary data source is the 13-item teacher questionnaire, complemented by structured observations of three students.

 Accordingly, we have removed the terminology “mixed-methods convergent parallel design” and no longer characterize the study as a convergent parallel mixed-methods study.

 The revised manuscript describes the study as a descriptive needs assessment within the initial phase of a broader Research and Development project, complemented by illustrative student observations.

 The student observations are now explicitly positioned as contextual and illustrative rather than as an independent qualitative strand used to establish methodological triangulation.

 We have also corrected the description in Section 2.3 so that it refers to the two actual data-collection approaches: questionnaire administration and structured student observation.

 Changes made: Abstract, Sections 2.1–2.3, Results, Discussion, and Conclusion.

**Comment 4. Psychometric evidence is incomplete**

 Report the actual Cronbach’s alpha (per dimension, noting that n = 20 makes it unstable), quantitative expert-validation results (CVI or Aiken’s V), and the full wording of all 13 items as an appendix (repisitory).

**Response**

 We appreciate this important suggestion.

 The revised manuscript now reports the internal-consistency analysis. The overall 13-item questionnaire yielded Cronbach’s α = 0.79. The dimension-level coefficients were approximately:

- Dimension 1: α = 0.65
- Dimension 2: α = 0.58
- Dimension 3: α = 0.18
- Dimension 4: α = 0.00

Because the questionnaire was administered to only 20 respondents and the dimensions contain only a small number of items, we now explicitly characterize these coefficients as exploratory and potentially unstable estimates rather than as evidence of a fully established psychometric instrument.

 The questionnaire was also reviewed by three experts with relevant expertise in mathematics education and inclusive education. Content validity was assessed using Aiken’s V. All 13 items obtained Aiken’s V values above the predetermined minimum criterion and were therefore retained as valid.

 Regarding the complete questionnaire wording, the full 13-item instrument is already openly available in the research repository associated with the study. We have therefore added an explicit statement directing readers to the repository rather than duplicating the instrument in an appendix.

 Changes made: Section 2.3, Data Availability Statement, and repository description.

**Comment 5. Questionnaire items are leading**

 The item “development of DIF-PBL is urgently needed” names the authors’ own model... Acknowledge acquiescence bias and this circularity in the Limitations.

**Response**

 We agree with this methodological concern.

 The revised manuscript now explicitly acknowledges that some questionnaire items are potentially leading or circular. In particular, Items 11–13 directly refer to the need for an integrated instructional model, an operational teacher guide, and the urgency of developing DIF-PBL. We also acknowledge that Item 10 incorporates the concrete–pictorial–symbolic progression directly into the questionnaire statement.

 Consequently, these responses are now interpreted as teachers’ perceived needs, rather than as independent empirical evidence establishing the effectiveness, superiority, or necessity of the proposed DIF-PBL model.

 We have also added acquiescence/framing bias to the limitations and explicitly acknowledged the researchers’ dual role as developers of the DIF-PBL model, which may have influenced questionnaire framing and interpretation.

 Changes made: Results, Section 4 (Limitations), and Conclusion.

**Comment 6. Root causes and traceability**

 The “Critical Root Cause” column restates the findings... describe the procedure or rename the column, add a traceability table, and reconcile the four requirements with the seven components...

**Response**

 We agree with the reviewer.

 The previous term “Critical Root Cause” suggested that a formal root-cause analysis had been conducted, although no such procedure was used. We have therefore replaced this terminology with “Underlying Instructional Factors” and removed claims implying formal causal analysis.

 We have also added a new Table 2: “Traceability from empirical findings to preliminary instructional requirements and DIF-PBL design implications.”

 The revised table explicitly maps:

 Empirical finding → Evidence source → Instructional requirement → Proposed DIF-PBL implication.

 This makes the relationship between the data and the subsequent design implications transparent.

 We have also clarified the distinction between the four questionnaire dimensions and the subsequent design implications. The four dimensions represent what was assessed in the questionnaire; they are not equivalent to the final design components.

 Furthermore, we agree that peer tutoring was not directly supported by a specific questionnaire item. It is therefore no longer presented as an empirically established requirement. If discussed, it is framed only as a possible implementation strategy.

 Adaptive assessment is treated differently because it is directly related to Item 4, which addresses the perceived inadequacy of current assessment instruments in accommodating differences in cognitive ability. Nevertheless, it is presented as a preliminary design implication, not as a validated DIF-PBL component.

 Changes made: Table 2, Abstract, Results, Discussion, and Conclusion.

**Comment 7. Student observations are under-reported**

 Specify grade level, type of SEN and how it was identified, definition of “slow learner”, numeracy tasks, sessions, observers and rubric; with n = 3, present these as illustrative cases.

**Response**

 We agree that the observations require more cautious and transparent reporting.

 The revised manuscript now explicitly describes the three observations as illustrative cases, rather than evidence from which population-level disparities can be inferred.

 The structured observation instrument covered four areas:

1. contextual problem solving;
2. mathematical representation, including concrete–pictorial–symbolic representation;
3. instructional support required; and
4. classroom participation.

The instrument used a four-point scale ranging from 1 = Very Limited to 4 = Independent, with observer notes for each indicator.

 The students represented three learner profiles: a regular student, a slow learner, and a student with special educational needs. The observations involved contextual numeracy tasks and focused on students’ approaches to problem solving, use of mathematical representations, instructional support requirements, and participation.

 Where detailed information such as formal diagnostic procedures, exact duration of each observation session, or formal classification criteria for “slow learner” was not systematically recorded in the dataset used for this article, we have not attempted to reconstruct or infer these details retrospectively. Instead, these limitations are now acknowledged explicitly, and the observations are treated as illustrative rather than generalizable evidence.

 Changes made: Sections 2.2–2.3, Section 3.5, Discussion, and Limitations.

**Comment 8. Sampling lacks detail**

 Report the number of schools and selection criteria, teacher characteristics... and how the teachers were recruited.

**Response**

 Thank you. We have expanded the participant and setting description.

 The revised manuscript specifies that the needs assessment involved three inclusive elementary schools. The schools were purposively selected because they implemented inclusive education in which regular students and students with special educational needs learned together in the same classrooms.

 The teacher questionnaire was completed by 20 teachers, consisting of classroom teachers and Special Education Support Teachers (GPK) involved in inclusive education.

 We have avoided adding teacher characteristics such as years of teaching experience, grade taught, specific inclusive-education training, or the exact number and type of SEN students when these characteristics were not systematically recorded in the dataset available for this article. Rather than inventing or retrospectively estimating these characteristics, the revised manuscript identifies their absence as a methodological limitation.

 Teacher participation was based on their direct involvement in teaching mathematics/numeracy in the participating inclusive classrooms.

 Changes made: Section 2.2 and Limitations.

**Comment 9. Check the citations**

 Several references do not support their claims... Add foundational sources and update AKM data.

**Response**

 We agree and have conducted a citation audit throughout the manuscript. The revised Introduction and Discussion have been checked to ensure that cited sources directly support the claims for which they are cited. In particular, we have reviewed the references concerning:

- inclusive numeracy education;
- Problem-Based Learning;
- differentiated instruction;
- learners with special educational needs;
- cognitive load and instructional scaffolding; and
- mathematical representation.

Where appropriate, foundational sources have been used directly rather than relying on secondary sources to support foundational theoretical claims. We have also reviewed the source used for national numeracy/AKM data and updated the relevant evidence where appropriate. References that did not directly support the associated claim have been removed or replaced.

 Changes made: Introduction, Discussion, and References.

**Comment 10. Structure**

 Rename Section 3 “Results”... and add a research question covering the “critical root causes.”

**Response**

 We agree with the first part of this comment. Section 3 has been renamed “Results”, while Section 4 remains “Discussion.”

 Regarding the proposed research question concerning “critical root causes,” we have not added a separate research question because the term has been removed from the manuscript. As noted above, the study did not conduct a formal root-cause analysis.

 Instead, the revised research questions focus on:

1. current conditions of numeracy learning;
2. challenges in implementing conventional PBL;
3. challenges in implementing differentiated instruction; and
4. preliminary instructional requirements emerging from the needs assessment.

This revised structure better corresponds to the actual design and analysis of the study.

 Changes made: Research Questions and Section 3 heading.

**Comment 11. Tone down the conclusions**

 “Comprehensive”, “evidence-based” and “replicable framework” go beyond what 20 perception questionnaires and three observations support; add self-report bias and the researchers’ dual role as model developers to the Limitations.

**Response**

 We agree. The revised manuscript has systematically moderated claims that could imply a stronger evidentiary basis than is warranted by the sample and exploratory design. Terms such as “comprehensive,” “evidence-based,” and “replicable framework” have been removed or qualified where appropriate.

 The conclusion now emphasizes that the needs assessment provides an empirical starting point and preliminary instructional basis for subsequent DIF-PBL development, rather than evidence of model effectiveness or superiority. We have also expanded the limitations to explicitly acknowledge:

- the small sample of 20 teachers;
- the three-school and single-district setting;
- reliance on teacher self-report data;
- potential acquiescence and framing bias in some questionnaire items;
- the limited number of student observations;
- the absence of a formal qualitative teacher strand; and
- the researchers’ dual role as developers of the DIF-PBL model.

Changes made: Sections 4.5–4.6 and Conclusion.

**Comment 12. Data and minor points**

 Share the raw observation records and a codebook, not only summaries; fix “The The”, the “Dr. Ranbir” citation format, the double comma in affiliation 1, and complete the CRediT roles.

**Response**

 We appreciate these detailed comments. The typographical and formatting issues identified by the reviewer have been corrected, including:

- the duplicated “The The”;
- the formatting of the “Dr. Ranbir” reference;
- the double comma in affiliation 1; and
- incomplete CRediT roles.

Regarding the observation data, the structured observation instrument and anonymized observation summaries are already available in the research repository. The observation instrument specifies the indicators, four-point rating scale, and observer-note structure. Because the observations involve only three students in specific school contexts, participant confidentiality was considered when preparing the shared materials. We have therefore ensured that identifying information is removed. 

 Changes made: Data Availability Statement, repository materials, References, affiliation, and CRediT statement.

**Overall response**

 We thank the reviewer for the constructive assessment and for recognizing the manuscript as a useful exploratory foundation for the subsequent development of the DIF-PBL model.

 In response to the review, we have substantially revised the manuscript to improve the alignment between the research design, data, analysis, and conclusions. In particular, we have:

1. corrected the interpretation of Item 8;
2. replaced the previous transformed percentage figures with item-level means, SDs, and actual response frequencies;
3. removed the overstated mixed-methods/convergent-parallel terminology;
4. reported the exploratory reliability analysis and clarified the questionnaire validation procedure;
5. acknowledged leading-item and acquiescence-bias concerns;
6. replaced “critical root causes” with more cautious terminology;
7. added a finding-to-requirement-to-design traceability table;
8. clarified the status of peer tutoring and adaptive assessment as design implications rather than uniformly empirically established components;
9. clarified the role and limitations of the three student observations;
10. expanded the description of the participating schools and teachers where the available data permit; and
11. moderated the conclusions and explicitly acknowledged self-report and researcher-role limitations.

The revised manuscript therefore positions the study more precisely as a descriptive needs assessment within the initial phase of a broader Research and Development project, with the resulting findings serving as a preliminary empirical basis for subsequent DIF-PBL design, validation, and effectiveness testing.

# Reviewer report: Reviewer response for version 1

**DOI:** 10.5256/f1000research.207432.r518276

**Published:** 2026-09-12

**Authors:** Andi Hasdiansyah

**Affiliations:** 1 Master of Pedagogy Program, Graduate School, Universitas Muhammadiyah Parepare, Parepare, Indonesia

**Overall assessment**

 The manuscript addresses a relevant and timely issue in inclusive numeracy education and provides a useful empirical foundation for the subsequent development of the DIF-PBL model. The progression from classroom needs assessment to instructional design requirements is logical, and the manuscript is generally clearly written and well organized. The use of teacher survey data complemented by observations of different learner profiles is appropriate for an exploratory needs-assessment study forming the initial stage of a broader Research and Development project.

 The manuscript also demonstrates good transparency by providing the questionnaire, anonymized teacher-response data, observation instrument, and observation summaries through an open repository.

 I have several points that should be addressed to improve methodological transparency and precision, but these do not, in my view, require substantial redesign of the study or additional data collection.

***First,*** the calculation and interpretation of the percentages presented in Figures 2–5 should be clarified. With 20 respondents, percentages such as 83.75%, 91.25%, and 93.75% cannot directly represent proportions of teachers. These values appear more likely to represent normalized Likert scores. The authors should clearly state the calculation formula and revise figure labels and textual interpretations where necessary.

***Second, *** some additional methodological details would improve reproducibility. Please report the exact reliability coefficient for the needs-assessment questionnaire, provide slightly more information regarding participant and school selection, clarify how the three student profiles were identified, and describe the procedure used to integrate questionnaire and observational evidence.

***Third, *** the term critical root cause should either be methodologically explained or replaced with more cautious terminology such as “underlying instructional factors” if a formal causal-analysis procedure was not undertaken.

**Fourth,** the relationship between the four broader design requirements described in the synthesis and the seven specific DIF-PBL components identified elsewhere should be clarified. A short mapping or explanatory paragraph would be sufficient.

***Finally, *** several references and citation details should be checked for accuracy and relevance, particularly where cited studies are used to support specific claims concerning inclusive numeracy, Problem-Based Learning, and learners with special educational needs.

 These revisions would improve the precision, transparency, and reproducibility of an otherwise useful exploratory study. I therefore consider the manuscript suitable for indexing after these relatively limited revisions are satisfactorily addressed.

Is the work clearly and accurately presented and does it cite the current literature?

Partly

If applicable, is the statistical analysis and its interpretation appropriate?

Partly

Are all the source data underlying the results available to ensure full reproducibility?

Yes

Is the study design appropriate and is the work technically sound?

Yes

Are the conclusions drawn adequately supported by the results?

Yes

Are sufficient details of methods and analysis provided to allow replication by others?

Partly

Reviewer Expertise:

Education

I confirm that I have read this submission and believe that I have an appropriate level of expertise to confirm that it is of an acceptable scientific standard, however I have significant reservations, as outlined above.

# Response

**Published:** 2026-10-01

**Authors:** Mentari Rama Putri

**Affiliations:** Postgraduate Program, State University of Makassar, Makassar, South Sulawesi, Indonesia

We sincerely thank the reviewer for the positive and constructive evaluation of our manuscript. We appreciate the recognition of the relevance of the topic, the logical progression from needs assessment to instructional design, and the transparency of the study materials through the open repository. We have carefully considered all of the reviewer’s comments and revised the manuscript to improve methodological precision, transparency, and reproducibility. Our responses are provided below.

 Comment 1. Clarification of the percentages in Figures 2–5

 The calculation and interpretation of the percentages presented in Figures 2–5 should be clarified. With 20 respondents, percentages such as 83.75%, 91.25%, and 93.75% cannot directly represent proportions of teachers. These values appear more likely to represent normalized Likert scores. The authors should clearly state the calculation formula and revise figure labels and textual interpretations where necessary.

 Response

 Thank you for highlighting this issue. We agree that the previous presentation could lead readers to interpret the reported values as proportions of teachers, which was not appropriate.

 To address this concern, we have removed Figures 2–5 and replaced them with a new item-level descriptive statistics table (Table 1). The revised table reports the mean, standard deviation, actual frequencies for Scores 1–4, and the proportion of respondents selecting Scores 3–4.

 The Results section has also been revised throughout. The previous wording referring to values such as 83.75% or 93.75% as the “percentage of teachers agreeing” has been removed. The revised manuscript now distinguishes between descriptive Likert-score statistics and actual response proportions. This allows readers to directly verify the findings from the response frequencies.

 Changes made: Results section and new Table 1.

 Comment 2. Additional methodological details

 Please report the exact reliability coefficient for the needs-assessment questionnaire, provide slightly more information regarding participant and school selection, clarify how the three student profiles were identified, and describe the procedure used to integrate questionnaire and observational evidence.

 Response

 We appreciate this suggestion and have expanded the Methods section accordingly.

 First, the internal consistency of the 13-item questionnaire was examined using Cronbach’s alpha. The overall questionnaire yielded α = 0.79. The dimension-level coefficients were approximately 0.65, 0.58, 0.18, and 0.00, respectively. Given the small sample size ( *n* = 20) and the small number of items within each dimension, we now explicitly characterize these dimension-level coefficients as exploratory and unstable estimates rather than as evidence of an established psychometric scale.

 Second, we have clarified the participant and site selection. The study involved 20 classroom teachers and Special Education Support Teachers (GPK) involved in inclusive elementary education. The research sites were purposively selected because they implemented inclusive education in which regular students and students with special educational needs learned together in the same classrooms.

 Third, the three student profiles were selected purposively to provide illustrative contrasts in learner characteristics: one regular student, one slow learner, and one student with special educational needs. The observation instrument was designed to document differences in contextual problem solving, mathematical representation, instructional support required, and classroom participation.

 Finally, we have clarified the procedure used to integrate the two sources of evidence. Questionnaire data were first analyzed descriptively to identify recurring teacher-reported instructional needs. Student observations were then analyzed descriptively across the three learner profiles. The two evidence sources were subsequently compared to identify recurring instructional issues, which were mapped to preliminary instructional requirements and design implications. We have revised the manuscript to describe this as an evidence-integration/traceability process, rather than overstating it as a formal mixed-methods triangulation design.

 Changes made: Sections 2.1–2.4, Results synthesis, and Table 2.

 Comment 3. Use of the term “critical root cause”

 The term critical root cause should either be methodologically explained or replaced with more cautious terminology such as “underlying instructional factors” if a formal causal-analysis procedure was not undertaken.

 Response

 We agree with the reviewer. The previous use of “critical root cause” implied that a formal causal-analysis procedure had been conducted, which was not the case.

 We have therefore replaced the term “critical root cause” with “underlying instructional factors” and removed references to formal root-cause analysis from the research aims and results synthesis.

 The revised analysis now focuses on the relationship between the empirical findings, evidence sources, instructional requirements, and preliminary DIF-PBL design implications. This terminology more accurately reflects the descriptive and exploratory nature of the present study.

 Changes made: Introduction, Results synthesis, Table 2, Discussion, and Conclusion.

 Comment 4. Relationship between four broader requirements and seven DIF-PBL components

 The relationship between the four broader design requirements described in the synthesis and the seven specific DIF-PBL components identified elsewhere should be clarified. A short mapping or explanatory paragraph would be sufficient.

 Response

 Thank you for identifying this inconsistency. We agree that the previous manuscript did not sufficiently distinguish between the four questionnaire dimensions, the instructional requirements emerging from the findings, and the specific design implications subsequently considered for DIF-PBL.

 We have addressed this issue by adding a new Table 2, “Traceability from empirical findings to preliminary instructional requirements and DIF-PBL design implications.” The table explicitly maps each empirical finding to its evidence source, corresponding instructional requirement, and proposed design implication.

 For example, differences in numeracy readiness identified through Items 1–3 and the illustrative observations are linked to the requirement for differentiated learning pathways and, subsequently, to tiered learning tasks. Similarly, Item 4 concerning the limited accommodation of learner differences in assessment informs the preliminary requirement for adaptive assessment and differentiated formative assessment.

 We have also revised the wording throughout the manuscript so that these are presented as preliminary instructional requirements and design implications, rather than as seven empirically validated DIF-PBL components.

 Importantly, the four dimensions of the questionnaire are now clearly distinguished from the resulting design implications. The four dimensions describe what was assessed, whereas the design implications describe how the findings may inform subsequent model development.

 Changes made: Results synthesis, new Table 2, Discussion, Abstract, and Conclusion.

 Comment 5. References and citation accuracy

 Several references and citation details should be checked for accuracy and relevance, particularly where cited studies are used to support specific claims concerning inclusive numeracy, Problem-Based Learning, and learners with special educational needs.

 Response

 We agree and have conducted a citation audit of the manuscript.

 The revised Introduction and Discussion now place greater emphasis on references that directly support the claims being made. Citations that did not adequately support specific statements concerning inclusive numeracy, Problem-Based Learning, differentiated instruction, or learners with special educational needs have been reviewed and replaced or removed where appropriate.

 We have also strengthened the use of foundational literature where appropriate, rather than relying exclusively on recent studies that may provide only indirect support for theoretical claims. Claims concerning the relevance of PBL, differentiated instruction, and learner diversity are now more carefully aligned with the scope of the cited evidence.

 Changes made: Introduction, Discussion, and reference list.

 Closing response

 We thank the reviewer for the constructive assessment and for recognizing the manuscript as a useful exploratory foundation for the subsequent development of DIF-PBL. In response to the comments, we have clarified the statistical presentation, expanded methodological reporting, replaced the unsupported “critical root cause” terminology, added a traceability framework linking empirical findings to preliminary design implications, and conducted a citation audit.

 These revisions also clarify the scope of the study: the present article reports a descriptive needs assessment as the initial phase of a broader Research and Development project, rather than an evaluation of the effectiveness of the DIF-PBL model. We believe the revisions have improved the methodological transparency, precision, and reproducibility of the manuscript.
